# Triple-isotope analysis in tree-ring cellulose suggests only moderate effects of tree species mixture on the climate sensitivity of silver fir and Douglas-fir

**DOI:** 10.1093/treephys/tpae067

**Published:** 2024-06-14

**Authors:** Justine Charlet de Sauvage, Kerstin Treydte, Matthias Saurer, Mathieu Lévesque

**Affiliations:** Silviculture Group, Institute of Terrestrial Ecosystems, ETH Zurich, Universitätstrasse 16, CH-8092 Zurich, Switzerland; Forest Dynamics, Swiss Federal Institute for Forest, Snow and Landscape Research WSL, Zürcherstrasse 111, CH-8903 Birmensdorf, Switzerland; Oeschger Centre for Climate Change Research, University of Bern, Hochschulstrasse 4, CH-3012 Bern, Switzerland; Forest Dynamics, Swiss Federal Institute for Forest, Snow and Landscape Research WSL, Zürcherstrasse 111, CH-8903 Birmensdorf, Switzerland; Silviculture Group, Institute of Terrestrial Ecosystems, ETH Zurich, Universitätstrasse 16, CH-8092 Zurich, Switzerland

**Keywords:** *Abies alba*, carbon isotope, drought, hydrogen isotope, oxygen isotope, *Pseudotsuga menziesii*

## Abstract

Disentangling the factors influencing the climate sensitivity of trees is crucial to understanding the susceptibility of forests to climate change. Reducing tree-to-tree competition and mixing tree species are two strategies often promoted to reduce the drought sensitivity of trees, but it is unclear how effective these measures are in different ecosystems. Here, we studied the growth and physiological responses to climate and severe droughts of silver fir and Douglas-fir growing in pure and mixed conditions at three sites in Switzerland. We used tree-ring width data and carbon (δ^13^C), oxygen (δ^18^O) and hydrogen (δ^2^H) stable isotope ratios from tree-ring cellulose to gain novel information on water relations and the physiology of trees in response to drought and how tree species mixture and competition modulate these responses. We found significant differences in isotope ratios between trees growing in pure and mixed conditions for the two species, although these differences varied between sites, e.g. trees growing in mixed conditions had higher δ^13^C values and tree-ring width than trees growing in pure conditions for two of the sites. For both species, differences between trees in pure and mixed conditions regarding their sensitivity to temperature, precipitation, climatic water balance and vapor pressure deficit were minor. Furthermore, trees growing in pure and mixed conditions showed similar responses of tree-ring width and isotope ratios to the past severe droughts of 2003, 2015 and 2018. Competition had only a significantly negative effect on δ^13^C of silver fir, which may suggest a decrease in photosynthesis due to higher competition for light and nutrients. Our study highlights that tree species mixture may have only moderate effects on the radial growth and physiological responses of silver fir and Douglas-fir to climatic conditions and that site condition effects may dominate over mixture effects.

## Introduction

Given the rapid changes in climate conditions and their impacts on forest ecosystems worldwide (Allen et al. 2015), it is a priority to better understand the growth and physiological responses of trees to climate change. However, these responses in uncontrolled environments are complex due to many interacting effects. For example, it is still unclear how tree species diversity and competition modulate the climate sensitivity of trees and if their modifications via forest management can reduce the drought susceptibility of trees.

Tree-ring width (TRW) is a valuable and widely used proxy to investigate tree responses to climate change, allowing the quantification of yearly stem radial growth over time (Fritts 1976). In addition, carbon (δ^13^C), oxygen (δ^18^O) and hydrogen (δ^2^H) stable isotope ratios in tree-ring cellulose are suitable indicators for inferring the physiological responses of trees to past biotic and abiotic effects (Siegwolf et al. 2022). These stable isotope ratios can provide information on water-use efficiency and gas exchange strategy of trees (McCarroll and Loader 2004).

The drivers of carbon and oxygen isotope fractionation in plant materials are well known (McCarroll and Loader 2004; Gessler et al. 2014). During water stress (either from competition and/or drought), δ^13^C and δ^18^O are expected to increase. While photosynthetic and stomatal conductance rates are reflected in tree-ring δ^13^C, δ^18^O mainly provides insights into the source water of the trees and transpiration or stomatal conductance rates at the leaf level (McCarroll and Loader 2004; Song et al. 2022). Although δ^2^H provides information about the source water, its signal is also modified by biochemical processes like the use of stored carbohydrates (Lehmann et al. 2022)*.* δ^18^O and δ^2^H in tree-ring cellulose carry different physiological and climatic signals (Vitali et al. 2022) and their decoupling under stress conditions could be related to increased carbohydrate storage use (Vitali et al. 2023). Analyzing δ^18^O and δ^2^H simultaneously can thus potentially provide complementary information on tree responses to climatic stress and how tree species mixture and competition modulate these responses.

So far, studies using stable isotopes in tree rings to analyze mixture effects on the response of trees to climate have focused on δ^13^C (e.g. Grossiord et al. 2014*b*; Bonal et al. 2017; Schwarz and Bauhus 2019) and also, though less, on δ^18^O (e.g. Vannoppen et al. 2020). None of them has, however, included δ^2^H measurements. Only recently, new analytical methods have made the measurement of δ^2^H in tree rings more accessible (Lehmann et al. 2022). The combined analysis of TRW, δ^13^C, δ^18^O and δ^2^H can provide complementary climatic and physiological information that is useful to understand the effects of tree species mixture on tree growth and climate sensitivity of trees.

The theory behind the expected positive effects of species diversity on tree growth and climate sensitivity is that trees growing in multispecific stands can benefit from reduced competition compared with trees growing in monospecific stands (competitive reduction; Forrester and Bauhus 2016). This competitive reduction can be explained by different root structures or temporal water use, i.e. through resource partitioning (Forrester and Bauhus 2016). However, this species diversity effect on tree radial growth response to drought is not always observed in natural environments where various external factors can modulate or hinder growth (Grossiord 2019; Haberstroh and Werner 2022).

Tree-ring isotopes can help to understand species diversity and competition effects on tree physiology. For example, Vannoppen et al. (2020) observed for European beech (*Fagus sylvatica* L.) that species diversity mitigated the drought response based on δ^13^C data, but the signal from δ^18^O was not sensitive to species diversity. The authors reported that during drought, the increase in δ^13^C was lower in beech trees growing in multispecific than monospecific stands, which indicated enhanced stomatal conductance and growth in mixtures. Competition, which we consider here as the interaction of individuals relying on the same resources, may be recorded in the isotope ratios since competition for light or water affects tree physiology, and hence isotope ratios (Marshall et al. 2022). Therefore, δ^13^C, δ^18^O and δ^2^H should be lower in trees growing in mixed conditions, where competitive reduction occurs, than in pure conditions with high intraspecific competition.

In this study, we focus on silver fir (*Abies alba* Mill.) and non-native Douglas-fir (*Pseudotsuga menziesii* (Mirb.) Franco), two important coniferous tree species promoted in Central Europe as a substitute for Norway spruce (*Picea abies* (L.) H. Karst.) due to their higher drought tolerance (Lévesque et al. 2014; Vitali et al. 2017; Vitasse et al. 2019). This drought tolerance is favored by the deep rooting system and the isohydric behavior of the two species (McMinn 1963; Bastien 2019; Vitasse et al. 2019). However, with the rapidly changing climatic conditions, the potential of silver fir and Douglas-fir to thrive in a warmer and drier climate is becoming uncertain (Vejpustková and Čihák 2019; Piedallu et al. 2023). Therefore, analyzing how these species responded to past severe droughts and understanding how competition and species diversity modulated this response are highly relevant for developing adaptive forest management strategies.

The main aim of our study was to analyze the growth and physiological responses to climate of silver fir and Douglas-fir in pure and mixed conditions. We asked the following research questions. (i) Are there differences in isotope ratios and TRW of silver fir and Douglas-fir between trees growing in pure and mixed conditions for the period 2000–2020? (ii) What are the responses of δ^13^C, δ^18^O, δ^2^H and TRW of silver fir and Douglas-fir growing in pure and mixed conditions to climate and past severe droughts? (iii) How does the local tree neighborhood (competition, tree species mixture and species diversity) influence the physiological responses and the radial growth response of silver fir and Douglas-fir to vapor pressure deficit?

## Materials and methods

### Data collection and dendrochronological methods

**Figure 1:**
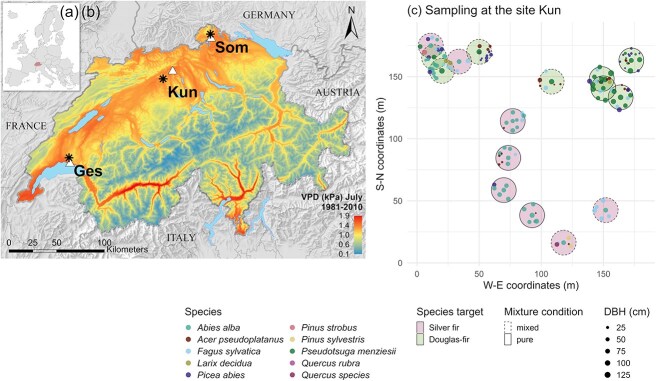
Map of (a) the location of Switzerland (red) within Europe (© EuroGeographics for the administrative boundaries) and of (b) the location of the three study sites in Switzerland. The white triangles indicate the location of the meteorological stations. Colors indicate the average VPD in July over the period 1981–2010, calculated based on the data of air temperature and relative air humidity provided by Huber et al. (2015) and based on MeteoSwiss data. The background map is from the Federal Office of Topography swisstopo. (c) Example of the sampling design of silver fir (pink shaded areas) and Douglas-fir (green shaded areas) at the site Kun (Küngoldingen). Each target tree is represented in the center of the 10 m radius circle and surrounded by its neighbor trees (colored dots, see the legend for the corresponding species). The dashed line and solid line circles represent the classification of the target trees as either in mixed or pure conditions, respectively.

We selected three sites in Switzerland based on the co-occurrence of mature silver firs and Douglas-firs, growing in pure and mixed conditions and with comparable age for a given species at a given site (Fig. 1, Tables 1 and 2). At each site, all the sampled trees irrespective of their group had comparable site conditions (i.e. soil, topography). At the three sites, Douglas-fir trees were planted in small groups or as single individuals in mixtures with other species originating from natural regeneration. Silver fir trees likely originate from natural regeneration and, as for Douglas-fir, grow in pure small groups or in mixtures with other species. In the last decades, all the stands have been managed according to close-to-nature silvicultural practices, consisting of low-intensity thinning interventions and continuous cover forestry. The climate of the three sites is temperate, with an average annual temperature between 9.5 and 9.9°C and an annual precipitation sum between 917 and 1094 mm for the period 2000–2020 (Table 1, Fig. S1 available as Supplementary data at *Tree Physiology* Online). Three severe summer droughts (i.e. 2003, 2015, and 2018) occurred during this period (Fig. S2 available as Supplementary data at *Tree Physiology* Online).

**Table 1:** Description of the sites with the climate variables for the period 2000–2020. The full names of the sites are given together with their three-letter abbreviation (used elsewhere in the text and figures). For latitude, longitude, and aspect of the slope, north (N), south (S), west (W) and east (E) refer to the cardinal points. The mean annual temperature, annual precipitation sum and CWB (based on April through September data) are given as mean ± standard deviation.

Site	Latitude (N)	Longitude (E)	Elevation (m a.s.l.)	Aspect	Slope (°)	Temperature (°C)	Precipitation (mm)	CWB (mm)
Bois des Gésiaux (Ges)	46° 33′ 20"	6° 39′ 12"	760	S	5	9.9 ± 6.4	1094 ± 219	6 ± 27
Küngoldingen (Kun)	47° 18′ 6"	7° 56′ 50"	480	W	8	9.8 ± 6.7	942 ± 144	−3 ± 20
Sommerwies (Som)	47° 43′ 6"	8° 36′ 41"	550	NE	10	9.5 ± 6.8	917 ± 137	−8 ± 18

**Table 2:** Description of the sampled trees. DBH, tree height, estimated age, TRW (tree-ring width of the period 2000–2020), competition (Hegyi index) and intraspecific competition are given with mean *±* standard deviation of four trees for each mixture group.

**Species**	**Site**	**Mixture**	**DBH (cm)**	**Tree height (m)**	**Est. age (years)**	**TRW (mm)**	**Competition (unitless)**	**Intraspecific competition (%)**
Silver fir	Ges	Pure	49.1 ± 2.9	27.4 ± 2.9	38 ± 7	7.36 ± 2.36	1.5 ± 0.1	88 ± 3
		Mixed	50.1 ± 3.8	26.0 ± 0.7	38 ± 10	5.54 ± 2.70	1.1 ± 0.4	8 ± 5
	Kun	Pure	68.7 ± 10.8	36.0 ± 3.3	103 ± 19	2.01 ± 1.23	0.8 ± 0.2	84 ± 7
		Mixed	72.3 ± 1.7	38.4 ± 2.2	92 ± 8	4.09 ± 3.07	0.6 ± 0.2	3 ± 6
	Som	Pure	39.4 ± 11.7	30.9 ± 3.9	46 ± 10	4.12 ± 2.02	1.8 ± 0.6	59 ± 10
		Mixed	56.7 ± 8.7	35.6 ± 1.6	66 ± 15	5.98 ± 3.32	0.8 ± 0.2	0 ± 0
Douglas-fir	Ges	Pure	99.1 ± 7.4	51.6 ± 1.8	103 ± 8	2.78 ± 1.04	0.8 ± 0.1	63 ± 7
		Mixed	109.9 ± 16.2	50.4 ± 3.4	105 ± 7	3.27 ± 2.14	0.7 ± 0.2	7 ± 7
	Kun	Pure	81.4 ± 12.6	50.7 ± 3.3	102 ± 9	3.56 ± 1.86	1.0 ± 0.2	55 ± 2
		Mixed	90.3 ± 11.7	48.8 ± 2.6	96 ± 7	4.69 ± 2.30	0.6 ± 0.2	0 ± 0
	Som	Pure	71.6 ± 8.6	48.0 ± 3.4	88 ± 15	3.45 ± 1.21	0.7 ± 0.2	82 ± 7
		Mixed	84.9 ± 16.8	43.7 ± 4.8	88 ± 19	4.06 ± 1.53	0.5 ± 0.1	19 ± 17

From the 20 silver fir and 20 Douglas-fir trees sampled at each study site in Charlet de Sauvage et al. (2023), we selected eight silver firs and eight Douglas-firs, all healthy and dominant/co-dominant (hereafter target trees) for isotope analysis. The trees were sampled between the end of August 2020 and May 2021. Trees were selected according to their neighborhood considering the level of tree species mixture. We selected the four individuals growing in the purest conditions (i.e. surrounded by their conspecifics) and the four individuals growing in the most mixed conditions (i.e. surrounded by trees from other species) for each species and at each site (see an example of sampling design in Fig. 1c and for all sites in the Supplementary data). Trees were carefully selected to be of similar age within a site and species to ensure comparability.

Two increment cores were sampled per tree at ca 50 cm from the ground and perpendicularly to the slope, with a 40- or 60-cm long increment borer (5.15 mm core diameter; Haglöf, Sweden). Tree cores were air dried, mounted on wooden holders, and sanded. Tree-ring widths were measured to the nearest 0.01 mm with either CooRecorder (v9.6, Cybis, Sweden) on scanned images or with TSAP-Win (v4.81) on a Lintab five measuring table (both from RINNTECH, Heidelberg, Germany). Both methods provided accurate and comparable measurements. Crossdating of TRWs was first performed visually and then confirmed statistically using COFECHA (Holmes 1983). We focused our analyses on the period 2000–2020 to include the dry years 2003, 2015 and 2018 and to study the most recent two decades with reliable neighborhood information since we measured the neighborhood in 2020 and used a static competition index (see Neighborhood Data). The diameter of the target trees (hereafter diameter) was reconstructed for the period 2000–2020 based on the diameter at coring height measured in the field and the TRWs. The age of the trees was estimated with the number of tree rings on the longest increment core for each tree plus the missing rings to the pith estimated with the software CooRecorder 9.6 (Cybis, Sweden).

### Sample preparation for isotope analysis

For each tree, we selected the core with the best quality (i.e. not broken, no missing rings, correctly crossdated) and with the highest correlation with the site chronology for isotope analysis. The tree cores, glued on wooden holders with wood glue (base of polyvinyl acetate, Geistlich, Switzerland), were soaked in hot water and detached from their support. The remaining glue was manually scraped off with a scalpel blade under a stereomicroscope. A simple test processing dried glue separately through the cellulose extraction steps revealed that the glue dissolved and was washed out during the chemical extraction so any glue residues on the wood samples would be removed.

Individual tree rings were split with a scalpel under a stereomicroscope into thin slices to facilitate cellulose extraction. We analyzed whole rings because Douglas-fir and silver fir have a gradual early- to latewood transition that makes the boundary between early- and latewood arbitrary. Additionally, the use of stored carbohydrates from previous years is of relatively minor importance for earlywood formation in conifers (Monson et al. 2018). Furthermore, the presence of narrow rings justified the use of the whole rings to ensure enough material for the isotope measurements. All tree rings were processed individually for the 48 trees and for each year for the period 2000–2020, leading to a total of 1008 samples.

The wood samples were packed into fiber filter bags (F57, Ankom Technology, USA), and the holocellulose (hereafter cellulose) was extracted following Boettger et al. (2007), modified according to Weigt et al. (2015). The cellulose extracted yielded 51.6 ± 4.1% and 54.5 ± 3.1% (mean ± standard deviation) of the original wood mass of the tree rings for silver fir and Douglas-fir, respectively. Cellulose samples were homogenized with an ultrasonic device (UP200S, Hielscher Ultrasonics, Germany) in ca 1 mL of distilled water, following Laumer et al. (2009). Samples were freeze-dried for up to 2 days (Beta 1–8 LD plus, Christ, Germany) to remove the water left from homogenization. Then, 1 ± 0.05 mg of cellulose was packed into silver capsules (3.3 × 5 mm, Säntis Analytical, Switzerland). To determine the hydrogen isotope ratios of carbon-bound hydrogen in the cellulose samples, the samples were equilibrated following procedures and calculations in Schuler et al. (2022). Finally, the samples were converted to H_2_ and CO by thermal decomposition at 1420 °C with a TC/EA (Pyrocube, Elementar, Hanau, Germany), and all isotope ratios were measured with an isotope-ratio mass spectrometer (MAT 253, Thermo, Germany). The precision of the analysis was 0.2‰ for δ^13^C and δ^18^O, and 1.5‰ for δ^2^H. Values of δ^13^C were corrected for changes in atmospheric δ^13^C (Belmecheri and Lavergne 2020) but not for changes in atmospheric CO_2_ concentration because this correction is too subjective (McCarroll et al. 2009; Treydte et al. 2009). This should not have biased our results because our study period was common to all the studied trees and covered only 21 years.

### Neighborhood data

Within a 10-m radius of each target tree, all the trees with a diameter at breast height (DBH) above 10 cm (hereafter neighbor trees) were measured. The DBH, species and distance to the target tree were recorded for each neighbor tree. The neighbor trees included, in descending order of abundance, silver fir, Norway spruce, Douglas-fir, European beech, sycamore maple (*Acer pseudoplatanaus* L.) and other less occurrent species (see Fig. 1c, Table S1 available as Supplementary data at *Tree Physiology* Online, and Supplementary data showing the maps of all the sampled trees). The distances between the target and neighbor trees were measured horizontally, from tree center to tree center, with a Vertex IV measuring device (Haglöf, Sweden).

To quantify the competition around each target tree, we calculated a distance-dependent competition index according to Hegyi (1974):


(1) \begin{equation*} {Competition}_t=\sum_{i=1}^n\frac{{DBH}_i\big/ {DBH}_t}{distance_{it}} \end{equation*}

with *t* referring to the target tree and *i* to the neighbor trees and their respective DBH. *n* is the number of neighbors for a given target tree. The *distance_it_* refers to the distance between the target tree and a given neighbor.

To evaluate the effects of intra- and inter-specific competition and quantify the degree of mixture, we calculated the percentage of intraspecific competition:


(2) \begin{align*} & \%{ Competition\ intraspecific}_t \nonumber \\ &\qquad =\frac{{Competition\ intraspecific}_t}{Competition_t}\times 100 \end{align*}

with *t* referring to the target tree. *Competition intraspecific_t_* is the competition calculated according to Eq. (1) including only the neighbor trees of the same species as the target tree. *Competition_t_* refers to the total competition of a target tree calculated according to the Eq. (1). Trees were classified as either in mixed or pure conditions (categorical variable) based on the percentage of intraspecific competition calculated with Eq. (2), if the value was below or above 50%, respectively.

To estimate the tree species diversity in the neighborhood of each target tree, we calculated the Shannon index (Shannon 1948) using the function *diversity* from the R package *vegan* (v2.6-2; Oksanen et al. 2022):


(3) \begin{equation*} {Species\ diversity}_t=-\sum_{i=1}^S{p}_i\cdotp \mathit{\ln}\left({p}_i\right) \end{equation*}

with $t$ referring to a given target tree, ${p}_i$ the proportional abundance of species *i* and *S* the number of species within the neighborhood of the target tree.

We focused the analyses on the period 2000–2020 because we measured the neighborhood in 2020–2021 and decided to consider a static neighborhood and competition instead of attempting to reconstruct the neighborhood back in time based on the TRW of the target trees. For easier reference, the variables used to characterize the neighborhood of the target trees are summarized in Table 3.

**Table 3:** Variables used to characterize the neighborhood of the target trees.

**Variable name**	**Reference**	**Definition**	**Variable type (with unit)**
Competition	Eq. (1)	Quantifies the total amount of competition around a target tree independently of the species.	Continuous (unitless)
Percentage of intraspecific competition	Eq. (2)	Portion of competition coming from intraspecific trees around the target tree. It is a percentage, thus the value does not reflect the absolute quantity of competition.	Continuous (%)
Mixed/pure	Based on the percentage of intraspecific competition	It is based on the percentage of intraspecific competition: mixed ≤ 50%; pure > 50%.	Categorical
Species diversity	Eq. (3)	It reflects the diversity of species around a target tree, including species richness and evenness.	Continuous (unitless)

### Climate data

Hourly average temperature, precipitation sum and relative humidity for the period 1999–2020 were retrieved from MeteoSwiss meteorological stations located near the study sites, which allowed us to calculate the daily minimum and maximum values. The stations were located 3 to 13 km from the study sites (Fig. 1, Table S2 available as Supplementary data at *Tree Physiology* Online). Temperature values were corrected for the differences in elevation between the sites and the meteorological stations with published monthly temperature lapse rates from Lotter et al. (2002).

Monthly vapor pressure deficit (VPD; kPa) was calculated with the monthly average of daily minimum and maximum temperature and relative humidity values (Eq. S1 available as Supplementary data at *Tree Physiology* Online). To answer research question (iii), we averaged the VPD for each year over the period April to September to include the entire growing period (Etzold et al. 2022; see also Response to climate and severe drought of trees in mixed and pure conditions). We chose to focus on VPD and not on other climatic variables in research question (iii) because VPD indicates the actual evaporative capacity of the atmosphere (Allen et al. 1998) and directly influences stomatal conductance (Grossiord et al. 2020), and thus isotopic variations in tree rings (McCarroll and Loader 2004).

We also estimated the climatic water balance (CWB; mm), which corresponds to precipitation minus potential evapotranspiration. The potential evapotranspiration was calculated from monthly average temperatures and the latitude of the sites following Thornthwaite (1948) with the R package *SPEI* (v1.8.1; Beguería and Vicente-Serrano 2023).

### Statistical analyses

To test the potential differences in isotope ratios and TRWs between pure and mixed trees (research question i), we performed Wilcoxon tests for each species, site and tree-ring variable (δ^13^C, δ^18^O, δ^2^H and TRW).

To evaluate the climate sensitivity of the species (research question ii), correlation analysis was performed between the tree-ring chronologies and seasonal climatic variables (VPD, temperature, precipitation sum and CWB) for the period 2000–2020. Ring-width indices (RWI) were obtained by applying a detrending with a cubic smoothing spline with a rigidity of 16 years on individual TRW series using the R package *dplR* (v1.7.4; Bunn et al. 2022). δ^13^C, δ^18^O and δ^2^H chronologies were calculated by averaging the yearly values per species, site and group. Seasonal climatic variables included 3-month averages, from June of the year before tree-ring formation to August of the year of tree-ring formation. Bootstrapped Pearson’s correlations were calculated with the function *dcc* from the R package *treeclim* (v2.0.6.0, Zang and Biondi 2015).

To assess the response of trees to drought (research question ii) and the potential differences in drought response between trees in pure and mixed conditions, we conducted superposed epoch analysis (SEA) based on the three driest years of the study period, i.e. 2003, 2015 and 2018 (Fig. S1 available as Supplementary data at *Tree Physiology* Online). SEA allowed us to evaluate the departure of tree-ring variables from mean values during superposed drought events. We ran the SEA with detrended and standardized chronologies of δ^13^C, δ^18^O, δ^2^H and RWI, with one chronology per species, site and mixture conditions. For this purpose, we applied the same spline detrending to the isotope series as for TRW. We restricted the analysis to 2 years prior to and after the drought to include the dry year 2018, which had only 2 years of post-drought data. The SEA was performed with a resampling of 1000 bootstrap samples to calculate the significance of the departure from the mean during and prior/after the drought year (*P* ≤ 0.05), using the function *sea* from the R package *dplR* (v1.7.4; Bunn et al. 2022).

To analyze the effects of competition, species diversity, tree species mixture, and their interaction with VPD (research question iii), we used linear mixed-effects models (one model per species and per tree-ring variable) using the function *lme* from the R package *nlme* (v3.1-157; Pinheiro et al. 2022):


(4) \begin{align*} {y}_{tj}=& \left({\beta}_0+{\mu}_{0, tj}\right)+{\beta}_1\cdot{diameter}_{tj}+{\beta}_2\cdot{VPD}_{Apr.- Sep{.}_j}\nonumber \\ & +{\beta}_3\cdot{species\ diversity}_t+{\beta}_4\cdot{competition}_t\nonumber \\ & +{\beta}_5\cdot \%{ competition\ intraspecific}_t\nonumber \\ & +{\beta}_6\cdot{species\ diversity}_t\cdot{VPD}_{Apr.- Sep{.}_j}\nonumber \\ & +{\beta}_7\cdot{competition}_t\cdot{VPD}_{Apr.- Sep{.}_j}\nonumber \\ & +{\beta}_8\cdot \%{ competition\ intraspecific}_t\cdot{VPD}_{Apr.- Sep{.}_j}+{\varepsilon}_{tj} \end{align*}

$$ {\varepsilon}_{tj}\sim N\left(0,{\sigma}^2\right),{\mu}_{0, tj}\sim N\left(0,{\sigma}_{\mu 0}^2\right) $$

where ${y}_{tj}$ is the response variable (δ^13^C, δ^18^O, δ^2^H or TRW) of a target tree *t* in year *j*, and the *β* are the coefficients. We log-transformed the response variable TRW and used non-detrended TRW series to retain the individual variability inherent to each tree. The years *j* included 2000 to 2020. *Diameter* refers to the reconstructed diameter of tree *t* in year *j* and is included in the model to account for tree size effect. *VPD_Apr.–Sep._* is the average VPD from April to September. In the results, we show fitted values for VPD values of 0.6, 0.7 and 0.8 kPa, which were in the range of our data (see Fig. S1c available as Supplementary data at *Tree Physiology* Online). *Species diversity*, *competition* and *% competition intraspecific* are described in Table 3. The normality of the errors ε and random effect μ_0_ (only calculated for the intercept) were assessed with visual plots. We did not include random slopes in the models because they prevented the models from converging. We tested for autocorrelation in our response variables and included an autocorrelation parameter of order 1 to account for temporal autocorrelation only for the model explaining TRW. We used tree identity as a grouping factor, nested in sites, for the random effect and the autocorrelation parameter. All predictor variables were scaled by subtracting their mean and dividing them by their standard deviation to allow comparison of effect sizes of the predictors.

Statistical analyses were performed with the software R (v4.2.1; R Core Team 2022), and all figures were plotted with *ggplot2* (v3.3.6, Wickham 2016), except for the map in Fig. 1 (ArcGIS Desktop v10.8).

## Results

### Differences in δ^13^C, δ^18^O, δ^2^H and TRW between trees in pure and mixed conditions

**Figure 2:**
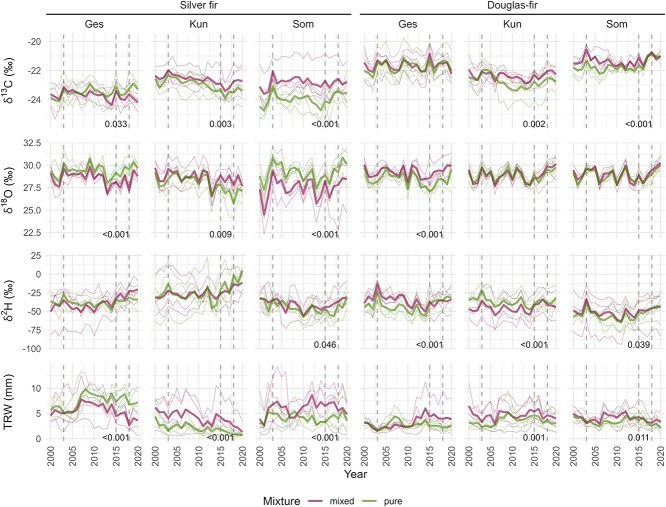
Time series of δ^13^C, δ^18^O, δ^2^H and TRW of silver fir and Douglas-fir growing in pure and mixed conditions at the three study sites (Ges: Bois des Gésiaux, Kun: Küngoldingen, Som: Sommerwies). Thick lines represent the average of four trees and thin lines the individual series of each tree. Significant differences (Wilcoxon test) between mixed and pure trees are indicated by *P*-values (≤0.05) at the bottom right corner of each subplot. For all *P*-values, see Table S3 available as Supplementary data at *Tree Physiology* Online. δ^13^C values were corrected for changes in atmospheric δ^13^C. Vertical dotted lines highlight the drought years 2003, 2015 and 2018 (see also Fig. S1 available as Supplementary data at *Tree Physiology* Online).

Silver fir and Douglas-fir trees growing in mixed conditions had significantly higher δ^13^C values than those in pure conditions at the sites Kun and Som (Fig. 2). In contrast, silver fir trees in pure conditions had significantly higher δ^13^C values than trees in mixed conditions at the site Ges, and there was no difference for Douglas-fir. δ^18^O in silver fir was significantly higher for trees growing in pure conditions than for trees in mixed conditions at the sites Ges and Som (Fig. 2). On the contrary, silver firs and Douglas-firs showed significantly higher δ^18^O values in mixed than in pure conditions at the sites Kun and Ges, respectively. δ^2^H values in silver fir were similar between pure and mixed conditions at the site Ges and Kun, whereas silver fir in pure conditions had lower δ^2^H values at the site Som (Fig. 2). For Douglas-fir, trees in mixed conditions had significantly higher δ^2^H values than those in pure conditions at the sites Ges and Som but lower values at the site Kun. Tree-ring width values were significantly higher for trees in mixed compared with pure conditions for both species at the sites Kun and Som (Fig. 2). At the site Ges, the TRW values of silver fir were lower in mixed than in pure conditions and not significantly different for Douglas-fir. At all sites and for both species, we observed the same significant differences for δ^13^C and TRW between trees in pure and mixed conditions. When δ^13^C values were higher in pure than in mixed conditions, the same was observed for TRW.

### Response to climate and severe drought of trees in mixed and pure conditions

**Figure 3:**
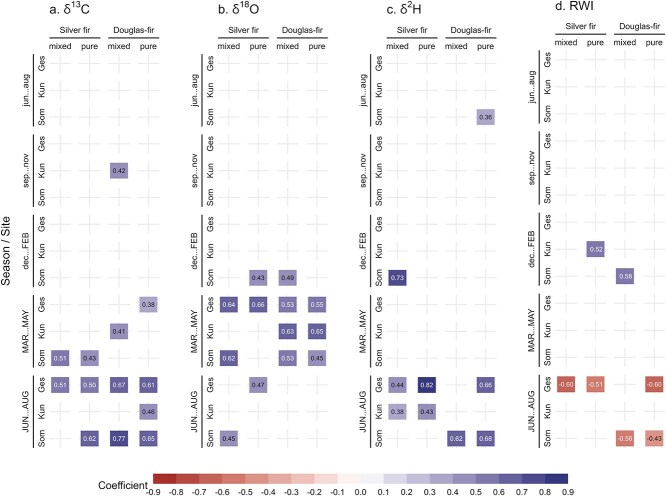
Bootstrapped Pearson’s correlation coefficients between mean seasonal VPD and tree-ring chronologies of (a) δ^13^C, (b) δ^18^O, (c) δ^2^H and (d) RWI for the period 2000–2020 at the three study sites (Ges, Kun, Som). Months in capital letters indicate months of the year of tree-ring formation. See Table 1 and Fig. 1 for the full names of the sites and their location. The colored cells with the values indicate significant correlations (*P*-value ≤ 0.05).

We found no clear differences in the sensitivity of all four tree-ring variables to temperature, precipitation, CWB, and VPD between trees growing in pure and mixed conditions (Fig. 3 and Supplementary Figs S3–S5 available as Supplementary data at *Tree Physiology* Online). Additionally, for both silver fir and Douglas-fir in pure and mixed conditions, the strength of the correlations varied among sites (the trees at the site Kun were the least climate-sensitive). Positive correlations occurred between δ^13^C and VPD in spring and particularly summer of the current year (Fig. 3a). δ^18^O correlated positively with VPD in spring, with Douglas-fir in pure and mixed conditions having significant correlations at all sites (Fig. 3b). δ^2^H correlated positively in summer, with correlation coefficients up to 0.82 for silver fir in pure conditions (Fig. 3c). RWI correlated negatively with VPD in summer of the current year (Fig. 3d).

**Figure 4:**
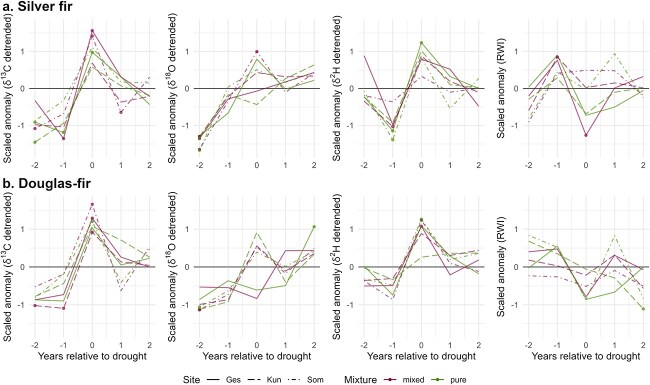
Superposed epoch analysis (SEA) for detrended δ^13^C, δ^18^O and δ^2^H and RWI of (a) silver fir and (b) Douglas-fir considering the drought years 2003, 2015 and 2018 (see Figs S1 and S2 available as Supplementary data at *Tree Physiology* Online). Significant departures (*P* ≤ 0.05) from 1000 random simulations are represented with filled circles.

For each tree-ring variable, the SEA of the drought years 2003, 2015, and 2018 revealed no clear differences between trees in pure and mixed conditions for silver fir and Douglas-fir (Fig. 4). During the drought years, the isotope ratios were generally high, particularly for δ^13^C and δ^2^H, and the RWI values were low. We observed stronger significant deviations from the mean for δ^13^C and δ^2^H compared with δ^18^O and RWI (Fig. 4). Most tree-ring variables were close to average values in the year following the severe droughts, although isotope ratios, particularly δ^18^O, remained slightly higher in the following 2 years after the drought events. In addition, RWI of Douglas-fir remained low after 2 years at some sites.

### Competition and species diversity effects on δ^13^C, δ^18^O, δ^2^H and TRW

**Figure 5:**
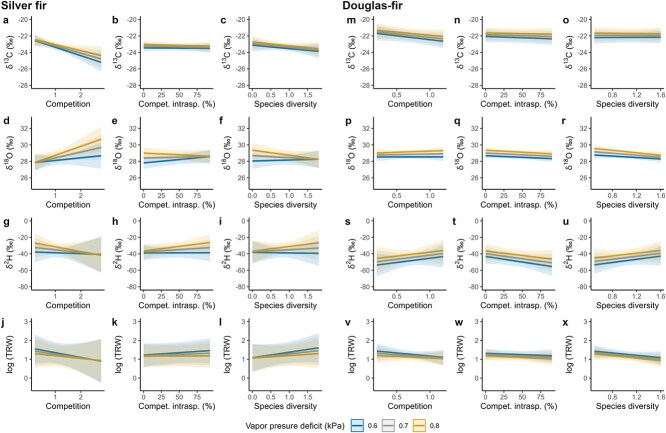
Effects of competition, percentage of intraspecific competition, and species diversity on δ^13^C, δ^18^O, δ^2^H and TRW of silver fir (a–l) and Douglas-fir (m–x) for the period 2000–2020. One model was used per response variable (i.e. δ^13^C, δ^18^O, δ^2^H and TRW) and per species following Eq. (4). The statistical outputs are shown in Table S4 available as Supplementary data at *Tree Physiology* Online. Vapor pressure deficit was included in the model as the average per year and per site for the period April to September. The fitted values are represented by the solid lines with 95% confidence intervals for moist (VPD = 0.6 kPa), intermediate (VPD = 0.7 kPa) and dry climatic conditions (VPD = 0.8 kPa).

Competition significantly and negatively influenced δ^13^C values of silver fir (Fig. 5a and Table S4 available as Supplementary data at *Tree Physiology* Online). We also observed some significant interactions between VPD and competition, between VPD and the percentage of intraspecific competition, or between VPD and species diversity for silver fir (Table S4 available as Supplementary data at *Tree Physiology* Online). For example, under dry climatic conditions (high VPD values) and high competition, δ^18^O values increased (Fig. 5d). Also, δ^18^O decreased under higher species diversity and drier conditions (Fig. 5f). δ^2^H of silver fir increased during drier conditions and for trees with rather pure neighborhoods (higher intraspecific competition; Fig. 5h). Apart from the main effect of competition on δ^13^C of silver fir, none of the main effects representing the neighborhood of the target trees (i.e. competition, percentage of intraspecific competition and species diversity) significantly influenced the tree-ring variables. For Douglas-fir, we found no significant main effect of competition, percentage of intraspecific competition, and species diversity nor significant interaction with VPD on the tree-ring variables (Fig. 5 and Table S4 available as Supplementary data at *Tree Physiology* Online). We only found a significant main effect of VPD on all tree-ring variables of Douglas-fir, also observed for silver fir.

## Discussion

### Differences in δ^13^C, δ^18^O, δ^2^H and TRW between trees in pure and mixed conditions

We found some significant differences in isotope ratios and TRW between trees growing in pure and mixed conditions, but the results were inconsistent across sites. At two out of the three studied sites (Kun and Som), silver fir and Douglas-fir trees growing in mixed conditions had higher δ^13^C values and TRW than trees growing in pure conditions, indicating higher photosynthetic rate and radial growth of the trees growing in mixed conditions. The higher TRW of the trees growing in mixed conditions could, however, also be due to the lower competition experienced by these trees in our study (Table 2).

Silver fir δ^18^O values in pure conditions were significantly higher than in mixed conditions at two of the three sites (Ges and Som). Assuming that δ^18^O in tree-ring cellulose is driven by the source water isotope signal modulated by leaf and soil water evaporative enrichment (Treydte et al. 2023), this suggests that silver fir in pure conditions likely relies more on a shallower soil water pool enriched in ^18^O (Treydte et al. 2014) than in mixed conditions. On the contrary, silver fir in mixed conditions would rely on a deeper soil water pool, which should, therefore, be an advantage during drought, as suggested by Gazol and Camarero (2016) and Lebourgeois et al. (2013). However, the tree-ring isotope signal is also influenced by leaf-level processes and by further re-exchange of oxygen atoms on the pathway from photosynthesis to wood formation, affecting the final δ^18^O signal in tree-ring cellulose (Gessler et al. 2013; Martínez-Sancho et al. 2023). Therefore, also differences in transpiration between trees growing in pure and mixed conditions could modify the δ^18^O values.

At first sight, the higher δ^2^H values of Douglas-fir trees in mixed conditions observed at two sites (Ges and Som) may indicate a preferential use of shallower soil water pools compared with trees in pure conditions since soil surface water tends to be more enriched in ^2^H in comparison to deeper soil horizons (Dawson et al. 2002). However, the opposite was observed at the third site (Kun). Mechanisms driving ^2^H fractionation until it is fixed in the tree rings are not yet fully understood (Lehmann et al. 2022), but there is clear evidence that tree-ring δ^2^H carries a strong physiological and biochemical signal besides source water signatures (Augusti et al. 2006; Lehmann et al. 2021; Vitali et al. 2022, 2023; Wieloch et al. 2022). Thus, higher δ^2^H values could indicate that trees in mixed conditions at the sites Ges and Som rely more on the use of ^2^H-enriched stored carbohydrates, while trees in pure conditions use more fresh ^2^H-depleted assimilates (Lehmann et al. 2021, 2022). Overall, the isotope ratios give some insights in photosynthetic rates (δ^13^C), source water uptake and rooting depth (δ^18^O), and use of stored carbohydrates (δ^2^H), although the differences between trees growing in mixed or pure conditions are not very clear.

### Response to climate and severe drought of trees in mixed and pure conditions

Previous studies suggested that the effects of tree species mixture on the drought response of trees range from negative to positive or even are absent depending on the drought severity (e.g. Haberstroh and Werner 2022). It is thus relevant to compare the climate sensitivity and response to severe drought of trees growing in pure and mixed conditions. Overall, we observed similar responses to VPD, temperature, precipitation and CWB between trees growing in pure and mixed conditions. The sensitivity of silver fir and Douglas-fir to interannual climate variability varied more among sites than between mixture conditions. Although we observed differences in isotope ratios between pure and mixed groups, as discussed in Differences in δ^13^C, δ^18^O, δ^2^H and TRW between trees in pure and mixed conditions, it does not necessarily imply that their sensitivity to interannual climate variability is also different. However, some studies, although based on TRW only and not stable isotopes, found that Douglas-fir in mixed conditions was less sensitive to climate than in pure conditions (Thurm et al. 2016). Similar results were observed for silver fir under extreme drought in Spain (Gazol and Camarero 2016).

Trees growing in mixed conditions could indeed be expected to be less affected by drought than trees growing in pure conditions due to enhanced resource-use efficiency and complementarity among species (Gazol and Camarero 2016; Thurm et al. 2016). This concept is based on the idea that species with contrasting functional traits may use resources differently in space and time. This is especially relevant when resources are limited, such as water during drought. For example, tree species with different root architecture can access water from different soil depths (Forrester and Bauhus 2016). In an experiment in Germany, Grossiord et al (2014*a*) analyzed the water uptake depth of young trees by spraying water labeled with deuterium on the soil surface during a dry summer. Young European beech, sessile oak (*Quercus petraea* (Matt.) Liebl.), Douglas-fir and Norway spruce were growing together, and the authors found that Douglas-fir trees did not show differences in the depth of soil water extraction in relation to the percentage of conifers in their neighborhood. This study found some differences only for European beech, with individuals growing within a higher percentage of conifers having a higher soil water extraction depth. Here, our two investigated species have taproots (McMinn 1963; Magh et al. 2020), allowing them to access water pools from deep soil horizons and rely less on surface water during drought. Although the differences in δ^18^O or δ^2^H time series between trees in pure and mixed conditions suggested some differences in the depth of main soil water access at some sites (Differences in δ^13^C, δ^18^O, δ^2^H and TRW between trees in pure and mixed conditions, Fig. 2), we did not observe a clear difference in the δ^18^O and δ^2^H responses to the severe 2003, 2015 and 2018 droughts between trees in pure and mixed conditions. Therefore, it is possible that under severe droughts, our investigated trees used a variety of soil water sources irrespective of their neighborhood conditions.

Although we did not find clear differences in climate sensitivity between trees in mixed and pure conditions, our results provide general insights into climate factors and seasonality driving the different tree-ring variables. Irrespective of the mixture conditions and tree species, we found that δ^18^O was significantly influenced by spring VPD or precipitation, whereas δ^2^H was significantly influenced by summer VPD or temperature. These seasonal differences support the often reported mismatch between δ^18^O and δ^2^H signals in tree rings (Lehmann et al. 2022) and emphasize the complementarity of δ^18^O and δ^2^H for inferring the climate response of trees. In addition, the drought signal in δ^13^C and δ^2^H, which may reflect a physiological response, was more pronounced than in δ^18^O and RWI (Fig. 4). The investigated severe droughts occurred in summer (see Fig. S2 available as Supplementary data at *Tree Physiology* Online) and we saw earlier that δ^13^C and δ^2^H were correlated to summer climatic conditions while δ^18^O was correlated to spring conditions (Fig. 3). This could explain why δ^13^C and δ^2^H showed a higher sensitivity to the summer droughts of 2003, 2015 and 2018 than δ^18^O and RWI in the SEA. Hartl-Meier et al. (2015) also observed a stronger summer drought signal in δ^13^C than in δ^18^O or TRW of Norway spruce, European beech and European larch (*Larix decidua* Mill.).

The results of the SEA further showed that silver fir and Douglas-fir mostly reached average values of δ^13^C, δ^18^O, δ^2^H and RWI in the year following the severe drought events irrespective of mixture conditions. This indicates that the physiology and growth of both species can overall recuperate after drought, as previously observed in comparable climatic regions by Lévesque et al. (2014) for Douglas-fir and Vitali et al. (2017) for silver fir and Douglas-fir. However, we still observed at some sites that 1 or 2 years after the drought some isotope values (and RWI) were still high (respectively low), indicating some lag effects from droughts.

### Competition and species diversity effects on δ^13^C, δ^18^O, δ^2^H and TRW

As opposed to research questions (i) and (ii), where we considered the mixture as a categorical variable (i.e. pure or mixed conditions), we considered tree species mixture as a continuous variable to answer research question (iii), by including the percentage of intraspecific competition and species diversity, and further included the competition effect in our linear mixed-effects models. For silver fir, we observed a significant negative effect of competition on δ^13^C (Fig. 5a). In general, δ^13^C increases (enrichment) when a tree experiences stressful conditions, as it is the case during drought and associated stomatal closure (Gagen et al. 2022). Following competition for resources, we would have then expected an increase in δ^13^C when neighborhood competition increases, but we found an opposite response. Although such a result seems counterintuitive at first sight, this response has also been reported for European beech (Mölder et al. 2011) and in a meta-analysis on the thinning effect on carbon isotope discrimination (Marshall et al. 2022). Tree-ring δ^13^C reflects changes in photosynthetic assimilation and stomatal conductance rates or water-use efficiency (McCarroll and Loader 2004). In our study, the observed decrease in δ^13^C under higher competition could be due to decreased photosynthetic assimilation rates because of competition for light and nutrients. Alternatively, the denser canopy cover likely associated with higher competition could limit evapotranspiration from the individual trees and mitigate their drought stress. Additionally, we observed a significant and positive interaction between competition and VPD on δ^13^C (Fig. 5a), which supports the results from Marshall et al. (2022), who found that the response of δ^13^C to lowered competition through thinning varied depending on the precipitation regime. In our case, the decrease in δ^13^C with increasing competition was stronger under moister climatic conditions (low VPD).

Regarding the effects of species diversity and mixture, we found several significant interactions between VPD and species diversity or the percentage of intraspecific competition for silver fir. From these interactions, it seemed that tree species mixture influenced the responses of δ^18^O, δ^2^H and TRW to VPD. However, only the interactions were significant (i.e. not the main effect), and the effects were small. For Douglas-fir, we found no significant effect of competition, species diversity or the percentage of intraspecific competition on tree-ring variables, indicating that the neighborhood of the trees did not influence their tree-ring isotope ratios nor TRW. Similar to the effects of tree species mixture on TRW (Grossiord 2019), the literature reports variable effects on isotope ratios. For example, Schwarz and Bauhus (2019) found no mixture effect on δ^13^C and drought resilience indices of silver fir and European beech at three sites in Germany and one site in Croatia. Vannoppen et al. (2020) observed a mixture effect on δ^13^C but not on δ^18^O in European beech in two temperate forests in Belgium. For Douglas-fir, the results from the literature are also variable. Thurm et al. (2016) found a reduced climate sensitivity for Douglas-fir in mixed stands, while Vitali et al. (2018) observed no effect of mixture on the radial growth of Douglas-fir during normal years but an increase in drought stress during dry years.

### Limitations of the study

We focused our analysis on the last 21 years of data (2000–2020) to control for important changes in stand conditions through time that could act as confounding factors. During the study period, the stand conditions (tree density) did not change much through management or natural mortality, as indicated by the visual assessment of the stumps during sampling. However, the stand conditions have likely changed throughout the ontogeny of the target trees, especially for older trees, and this might have influenced, for example, the root development and soil water uptake source of the trees over time. Information about past forest management and natural mortality at the time of tree establishment that may have influenced the stand dynamics around the target trees could have been helpful but was unavailable.

We sampled trees of different age classes across sites and species. For example, at the sites Ges and Som, silver fir and Douglas-fir trees did not have the same age. Although these age differences imply that the two species have established at different times within a site, the silver firs and Douglas-firs growing in mixed conditions did not necessarily grow together. Any other species could surround them (see details of the sampling in the Supplementary data). Since we did not directly compare the two species within a site but rather the groups of trees growing in pure and mixed conditions within a species, the age differences did not cause bias in our comparison.

## Conclusions

We used a triple-isotope approach and TRW measurements to better understand the growth and physiological responses of silver fir and Douglas-fir to climate and drought and how tree species mixture and competition modulate these responses. We found that the effects of tree species mixture and competition on tree-ring variables varied between species and among sites. Our results highlight that tree species mixture had only a weak or no effect on climate and drought sensitivity of Douglas-fir and silver fir, possibly because other confounding and uncontrolled factors (e.g. microsite conditions, belowground competition, species identity) can interplay and mask such mixture effects. To evaluate the effects of mixing different tree species, it might be insightful to look at specific species combinations to precisely test the expected benefits of mixture types on drought sensitivity. From a physiological point of view and at the individual tree level, we found few differences between trees growing in pure and mixed conditions. However, studying these effects at the population level might result in different response patterns of mixed and pure stands to drought. Finally, although our study did not find a lower drought sensitivity of trees in mixed conditions, promoting diverse tree species and favoring mixed forests are still efficient silvicultural options to lower the risk of pest attacks (Brockerhoff et al. 2017), promote the conservation of forest biodiversity (Cavard et al. 2011) and sustainably provide a wide range of ecosystem services.

## Supplementary Material

Supplementary_Data_figures_tables_tpae067

Supplementary_Data_maps_sampling_tpae067

## Data Availability

The dataset is available at doi: 10.16904/envidat.527 (Charlet de Sauvage et al. 2024).
