# Supplementary material for: Triple-isotope analysis in tree-ring cellulose suggests only moderate effects of tree species mixture on the climate sensitivity of silver fir and Douglas-fir
Source: Tree Physiol. 2024 Jun 14;44(7):tpae067. doi: 10.1093/treephys/tpae067 (PMC11247184; doi:10.1093/treephys/tpae067)
Supplement: Supplementary_Data_figures_tables_tpae067 [file supplementary_data_figures_tables_tpae067.docx]

**Triple-isotope analysis in tree-ring cellulose suggests only moderate effects of tree species mixture on the climate sensitivity of silver fir and Douglas-fir**

Justine Charlet de Sauvage, Kerstin Treydte, Matthias Saurer, Mathieu Lévesque

**Supplementary Data**

**Supplementary table 1.** Average percentages of competition from each neighbor species per site, species and mixture condition. The totals vary slightly from 100 because the numbers are rounded for easier reading.

| **Site** | **Species target** | **Mixture** | *Abies alba* | *Pseudotsuga menziesii* | *Alnus glutinosa* | *Acer platanoides* | *Acer pseudoplatanus* | *Betula pendula* | *Corylus avellana* | *Castanea sativa* | *Fraxinus excelsior* | *Fagus sylvatica* | *Larix decidua* | *Picea abies* | *Prunus avium* | *Pinus strobus* | *Pinus sylvestris* | *Quercus petraea* | *Quercus rubra* | *Quercus species* | *Sorbus aucuparia* | *Salix species* | *Taxus baccata* | *Tilia cordata* | Total |
| --- | --- | --- | --- | --- | --- | --- | --- | --- | --- | --- | --- | --- | --- | --- | --- | --- | --- | --- | --- | --- | --- | --- | --- | --- | --- |
| Ges | *A. alba* | mixed | 8 | 48 | 0 | 0 | 18 | 0 | 0 | 0 | 4 | 0 | 0 | 4 | 2 | 0 | 10 | 2 | 0 | 0 | 1 | 2 | 1 | 0 | 100 |
| Ges | *A. alba* | pure | 88 | 0 | 0 | 0 | 2 | 0 | 0 | 0 | 0 | 0 | 0 | 3 | 0 | 0 | 0 | 4 | 0 | 0 | 0 | 2 | 0 | 0 | 99 |
| Ges | *P. menziesii* | mixed | 63 | 7 | 10 | 0 | 2 | 0 | 1 | 0 | 2 | 2 | 0 | 2 | 5 | 0 | 0 | 0 | 0 | 0 | 4 | 2 | 0 | 0 | 100 |
| Ges | *P. menziesii* | pure | 20 | 62 | 0 | 0 | 7 | 0 | 0 | 0 | 0 | 2 | 0 | 4 | 3 | 0 | 0 | 0 | 0 | 0 | 0 | 0 | 0 | 0 | 98 |
| Kun | *A. alba* | mixed | 4 | 4 | 0 | 0 | 2 | 0 | 0 | 0 | 0 | 54 | 8 | 10 | 0 | 0 | 12 | 0 | 0 | 8 | 0 | 0 | 0 | 0 | 102 |
| Kun | *A. alba* | pure | 84 | 0 | 0 | 0 | 6 | 0 | 0 | 0 | 0 | 6 | 0 | 3 | 0 | 0 | 0 | 0 | 0 | 0 | 0 | 0 | 0 | 0 | 99 |
| Kun | *P. menziesii* | mixed | 32 | 0 | 0 | 0 | 18 | 0 | 0 | 0 | 0 | 17 | 3 | 12 | 0 | 5 | 0 | 0 | 13 | 0 | 0 | 0 | 0 | 0 | 100 |
| Kun | *P. menziesii* | pure | 0 | 55 | 0 | 0 | 10 | 0 | 0 | 0 | 0 | 16 | 2 | 17 | 0 | 0 | 0 | 0 | 0 | 0 | 0 | 0 | 0 | 0 | 100 |
| Som | *A. alba* | mixed | 0 | 0 | 0 | 0 | 14 | 0 | 0 | 0 | 0 | 14 | 0 | 63 | 0 | 0 | 4 | 0 | 0 | 0 | 0 | 0 | 0 | 5 | 100 |
| Som | *A. alba* | pure | 59 | 0 | 0 | 0 | 2 | 0 | 0 | 0 | 0 | 0 | 1 | 34 | 0 | 0 | 0 | 0 | 0 | 0 | 0 | 0 | 0 | 4 | 100 |
| Som | *P. menziesii* | mixed | 0 | 19 | 0 | 3 | 4 | 0 | 0 | 0 | 0 | 28 | 3 | 26 | 0 | 0 | 0 | 14 | 0 | 0 | 0 | 0 | 0 | 4 | 101 |
| Som | *P. menziesii* | pure | 0 | 82 | 0 | 0 | 0 | 0 | 0 | 0 | 0 | 9 | 0 | 2 | 0 | 0 | 0 | 0 | 0 | 0 | 0 | 0 | 0 | 6 | 99 |

**Supplementary Table 2.** Description of the meteorological stations used for the climate data. Source of the data: MeteoSwiss.

| **Site** | **Station** | **Latitude (N)** | **Longitude (E)** | **Elevation**  **(m a.s.l.)** | **Distance to site (km)** |
| --- | --- | --- | --- | --- | --- |
| Ges | Pully | 46° 30′ 44.218″ | 6° 40′ 03.058″ | 456 | 5.0 |
| Kun | Buchs - Aarau | 47° 23′ 03.763″ | 8° 04′ 46.341″ | 386 | 13.5 |
| Som | Schaffhausen | 47° 41′ 23.401″ | 8° 37′ 12.571″ | 438 | 3.2 |

**Supplementary Equation 1**

We calculated the monthly vapor pressure deficit (VPD; kPa) after Eq. 11 and 17 in Allen et al. (1998):

$$VPD=SVP-AVP$$

with SVP (saturated vapor pressure; kPa):

$$SVP=({SVP}_{Tmin}+{SVP}_{Tmax})/2$$

with SVP_Tmin_ the saturated vapor pressure at daily minimum temperature (monthly average):

$${SVP}_{Tmin}=0.6108\cdot\exp\left( \frac{17.27\cdot T_{min}}{T_{min}+237.3} \right)$$

and SVP_Tmax_ the saturated vapor pressure at daily maximum temperature (monthly average):

$${SVP}_{Tmax}=0.6108\cdot\exp\left( \frac{17.27\cdot T_{max}}{T_{max}+237.3} \right)$$

and with AVP (actual vapor pressure; kPa):

$$AVP=\left[ {SVP}_{Tmin}\cdot\frac{{RH}_{max}}{100}+{SVP}_{Tmax}\cdot\frac{{RH}_{min}}{100} \right]/2$$

with T_min_ (T_max_) the monthly average daily minimum (maximum) temperature (°C) and RH_min_ (RH_max_) the average monthly daily minimum (maximum) relative humidity (%).


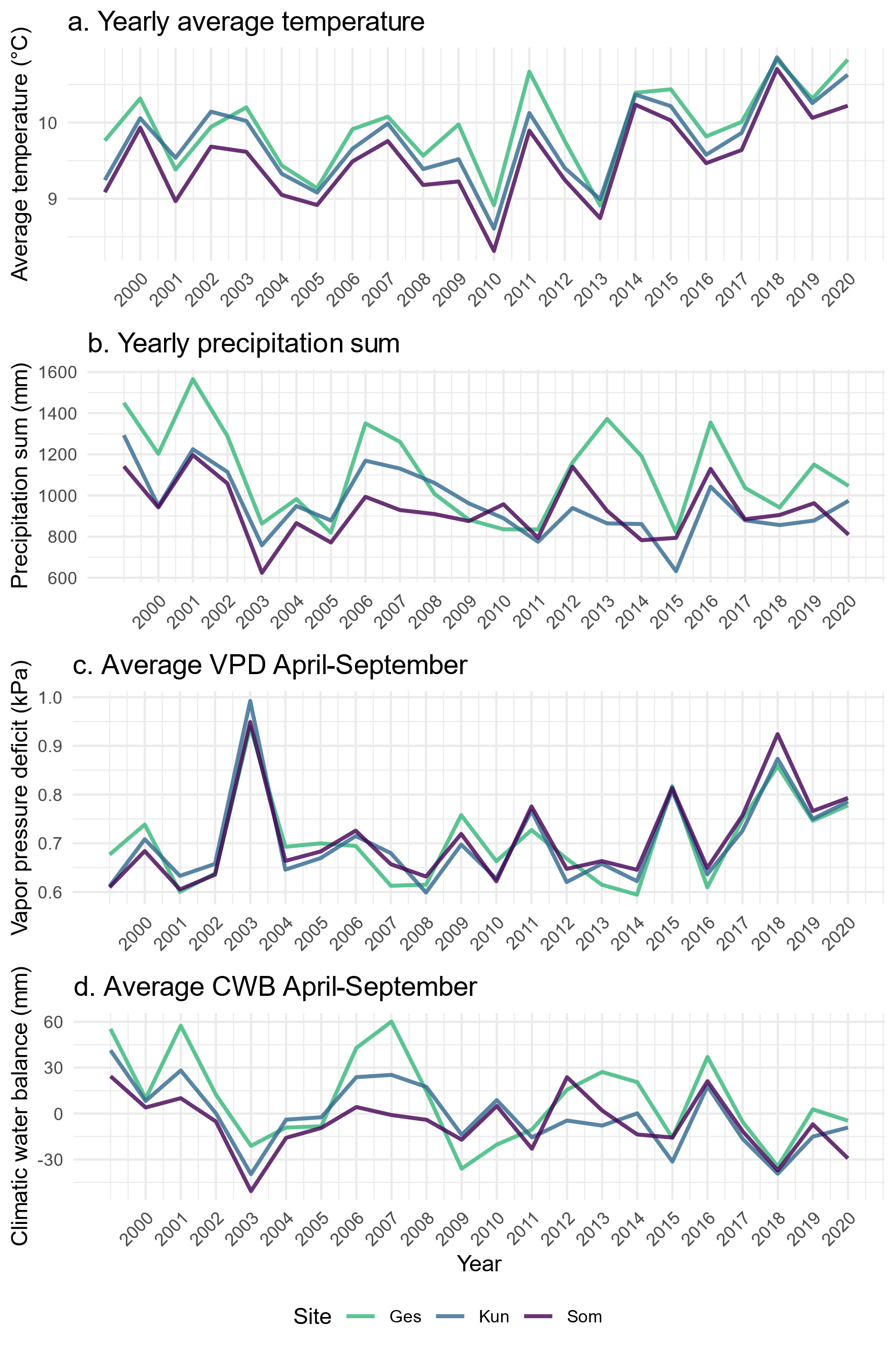


**Supplementary Figure 1.** Yearly average temperature (a), yearly precipitation sum (b), average vapor pressure deficit (VPD; c) and climatic water balance (CWB; d) for the period April to September at the three sites and for the period 2000–2020. For the full name of the sites and their locations, see Table 1 and Figure 1. VPD was calculated following Supplementary Equation 1.


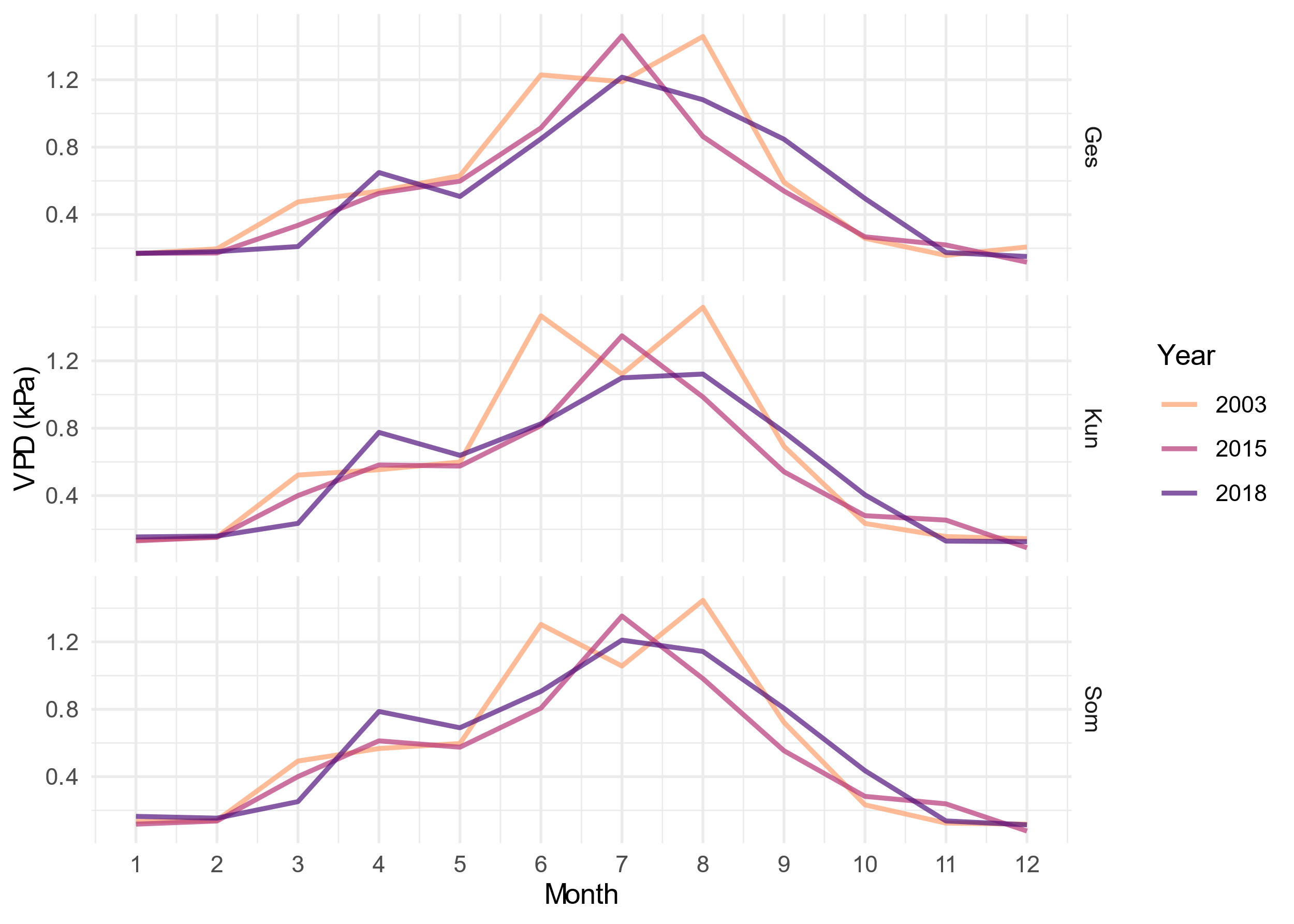


**Supplementary Figure 2.** Monthly vapor pressure deficit (VPD) at the three study sites during the three drought years selected for the analyses. The full names of the sites are given in Table 1.

**Supplementary Table 3.** Wilcoxon tests between pure and mixed groups of trees per species and site for (a) δ^13^C (b) δ^18^O (c) δ^2^H and (d) TRW. The time series are shown in Figure 2. For each comparison, 84 observations are included in each group (i.e., 4 trees with 21 years of observation each, per group pure and mixed).

| **(a) δ^13^C** | | |
| --- | --- | --- |
| **Site** | **Species** | **P-value** |
| Ges | Silver fir | 0.033 |
| Ges | Douglas-fir | 0.337 |
| Kun | Silver fir | 0.003 |
| Kun | Douglas-fir | 0.002 |
| Som | Silver fir | <0.001 |
| Som | Douglas-fir | <0.001 |
|  | | |
| **(b) δ^18^O** | | |
| **Site** | **Species** | **P-value** |
| Ges | Silver fir | <0.001 |
| Ges | Douglas-fir | <0.001 |
| Kun | Silver fir | 0.009 |
| Kun | Douglas-fir | 0.971 |
| Som | Silver fir | <0.001 |
| Som | Douglas-fir | 0.279 |
|  | | |
| **(c) δ^2^H** | | |
| **Site** | **Species** | **P-value** |
| Ges | Silver fir | 0.24 |
| Ges | Douglas-fir | <0.001 |
| Kun | Silver fir | 0.6 |
| Kun | Douglas-fir | <0.001 |
| Som | Silver fir | 0.05 |
| Som | Douglas-fir | 0.04 |
|  | | |
| **(d) TRW** | | |
| **Site** | **Species** | **P-value** |
| Ges | Silver fir | <0.001 |
| Ges | Douglas-fir | 0.722 |
| Kun | Silver fir | <0.001 |
| Kun | Douglas-fir | 0.001 |
| Som | Silver fir | <0.001 |
| Som | Douglas-fir | 0.011 |


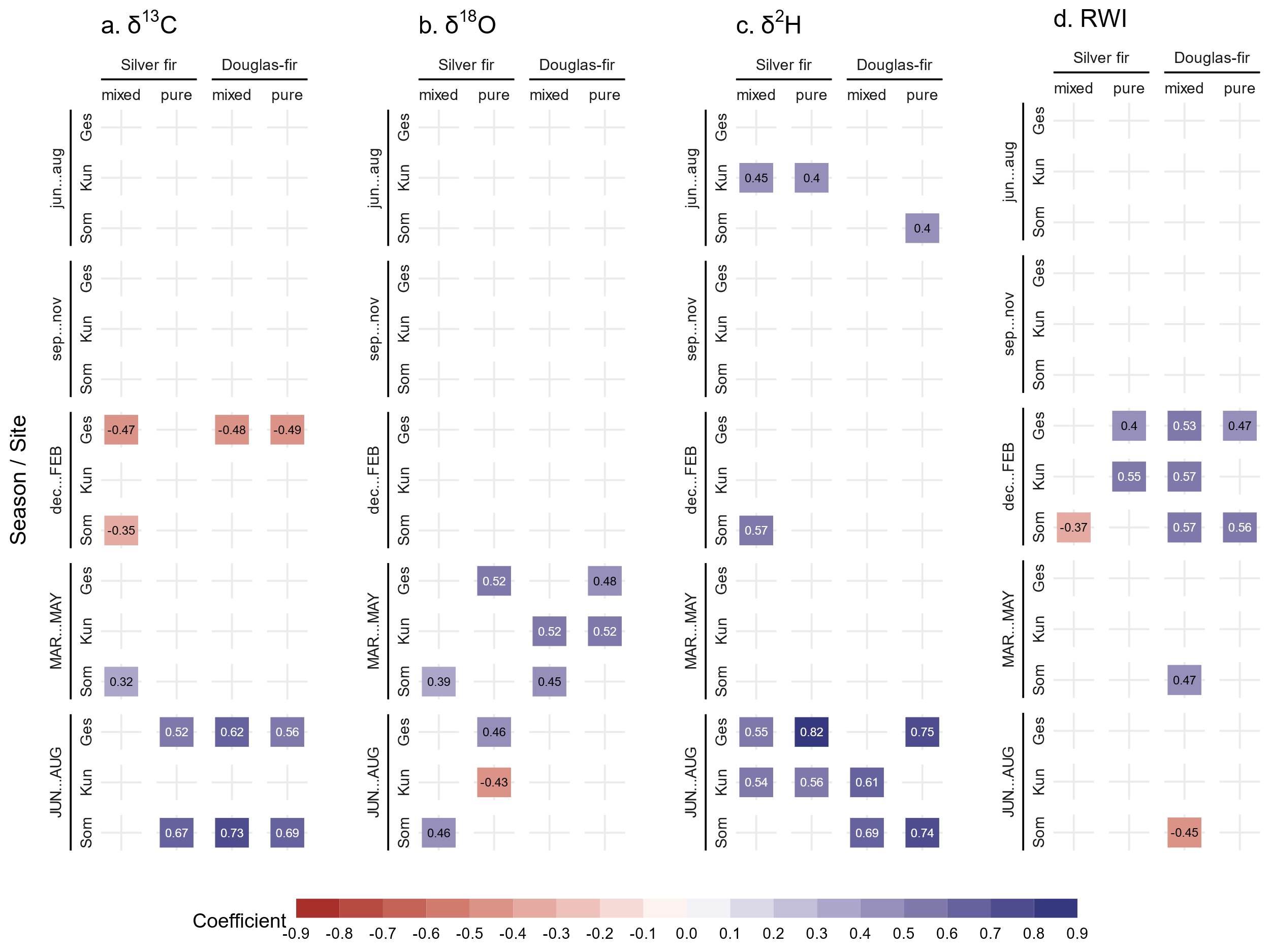


**Supplementary Figure 3.** Bootstrapped Pearson’s correlation coefficients between mean seasonal temperature and tree-ring chronologies of: (a) δ^13^C, (b) δ^18^O, (c) δ^2^H and (d) ring-width indices (RWI) for the period 2000–2020 at the three study sites (Ges, Kun, Som). Months in capital letters indicate months of the year of tree-ring formation. See Table 1 and Figure 1 for the full names of the sites and their location. The colors and the values in each cell indicate significant correlations (P-value ≤ 0.05).


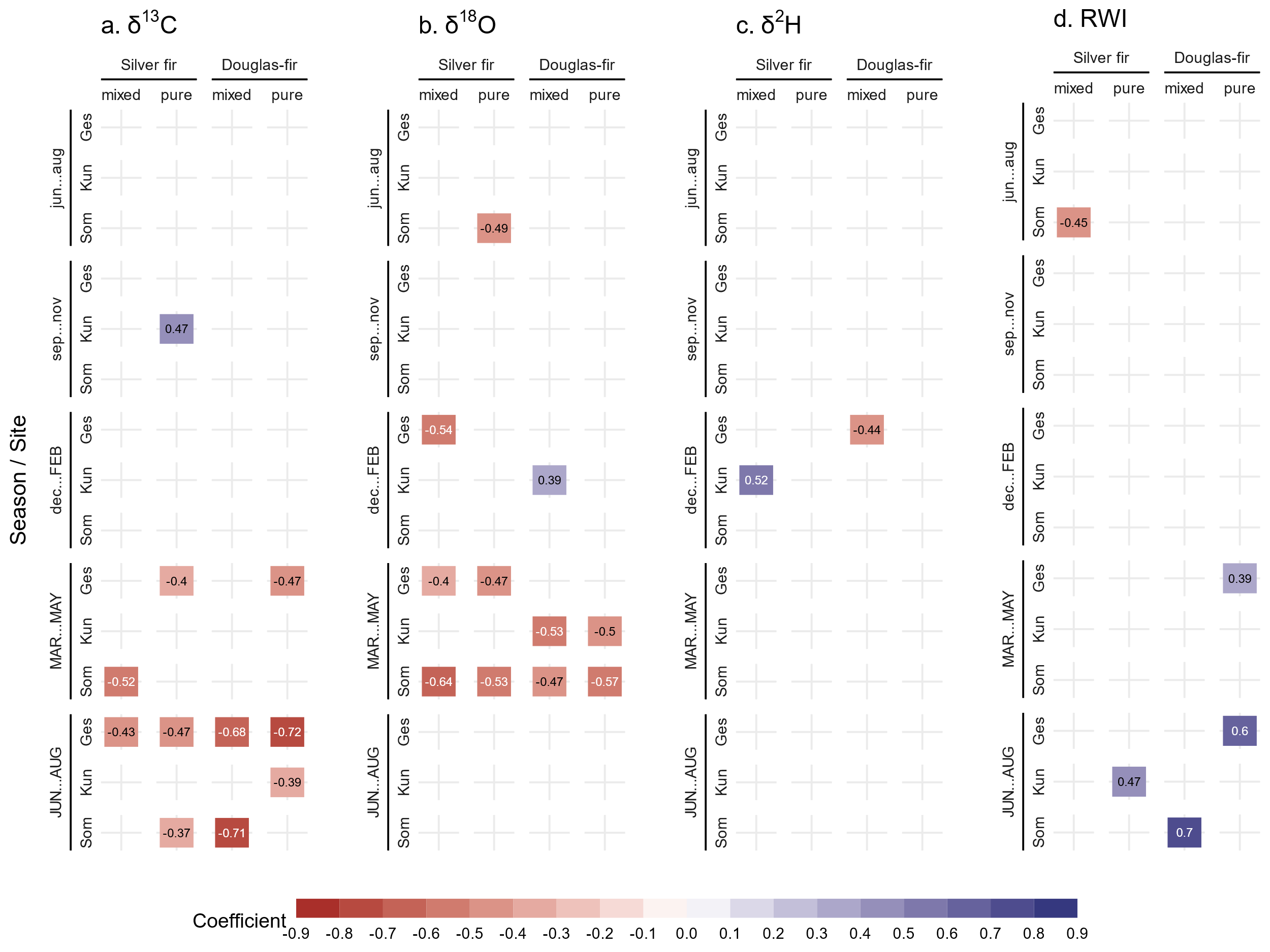


**Supplementary Figure 4.** Bootstrapped Pearson’s correlation coefficients between seasonal precipitation sum and tree-ring chronologies of: (a) δ^13^C, (b) δ^18^O, (c) δ^2^H and (d) ring-width indices (RWI) for the period 2000–2020 at the three study sites (Ges, Kun, Som). Months in capital letters indicate months of the year of tree-ring formation. See Table 1 and Figure 1 for the full names of the sites and their location. The colors and the values in each cell indicate significant correlations (P-value ≤ 0.05).


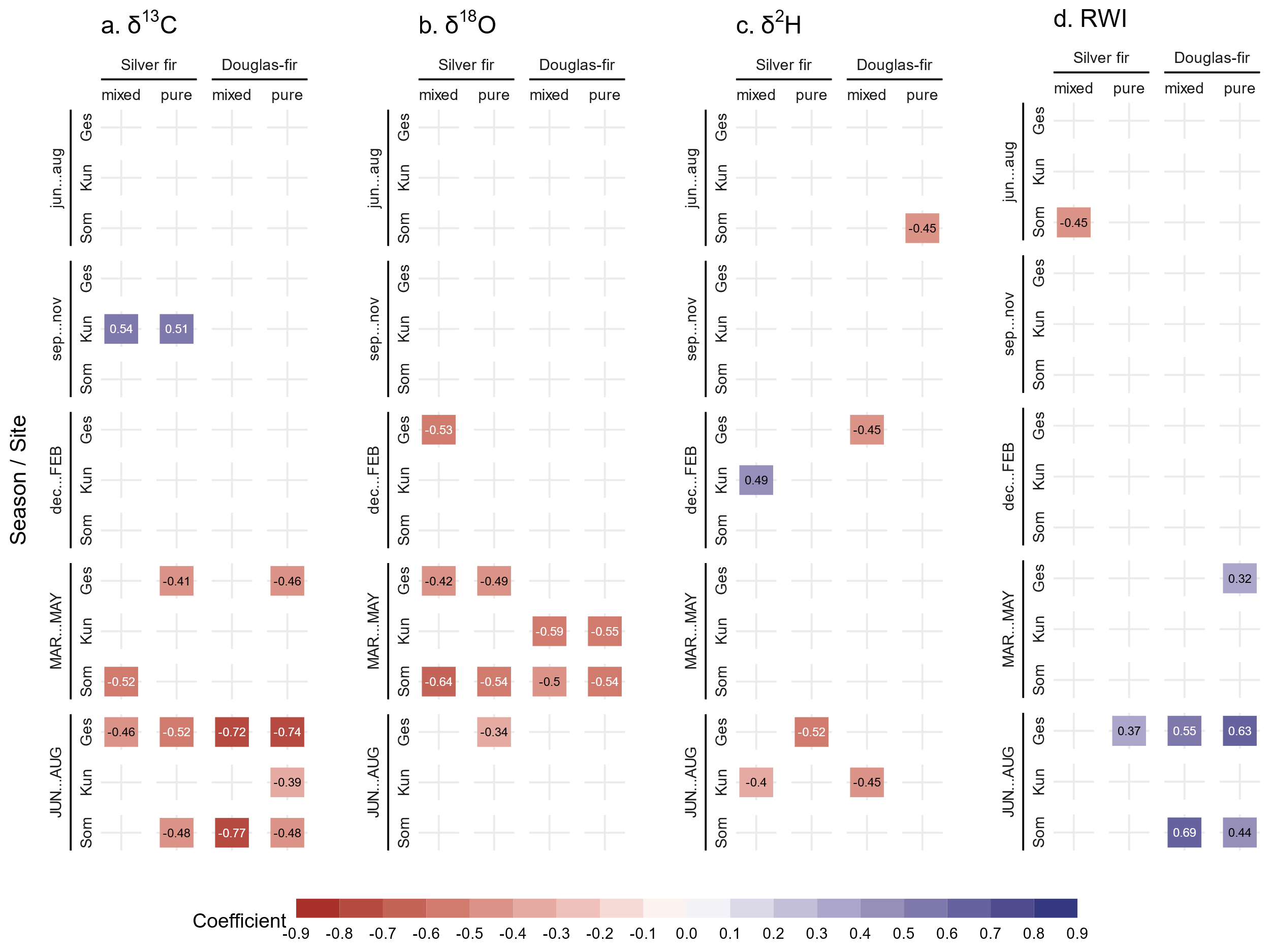


**Supplementary Figure 5.** Bootstrapped Pearson’s correlation coefficients between mean seasonal climatic water balance and tree-ring chronologies of: (a) δ^13^C, (b) δ^18^O, (c) δ^2^H and (d) ring-width indices (RWI) for the period 2000–2020 at the three study sites (Ges, Kun, Som). Months in capital letters indicate months of the year of tree-ring formation. See Table 1 and Figure 1 for the full names of the sites and their location. The colors and the values in each cell indicate significant correlations (P-value ≤ 0.05).

**Supplementary Table 4.** Statistical summary of the mixed-effects models shown in Figure 5 and calculated following equation (4). VPD stands for vapor pressure deficit. Significant P-values (≤ 0.05) are highlighted in bold.

| **Silver fir** | | | | |
| --- | --- | --- | --- | --- |
| **Response variable** | **Predictor variable** | **Estimate** | **Standard error** | **P-value** |
| δ^13^C | Intercept | -23.278 | 0.140 | **<0.001** |
|  | Diameter | -0.114 | 0.061 | 0.061 |
|  | VPD | 0.167 | 0.021 | **<0.001** |
|  | Competition | -0.475 | 0.172 | **0.013** |
|  | % Competition intrasp. | -0.061 | 0.172 | 0.729 |
|  | Species diversity | -0.176 | 0.162 | 0.292 |
|  | VPD:Competition | 0.069 | 0.024 | **0.005** |
|  | VPD:% Competition intrasp. | -0.030 | 0.025 | 0.234 |
|  | VPD:Species diversity | -0.006 | 0.024 | 0.807 |
| δ^18^O | Intercept | 28.522 | 0.279 | **<0.001** |
|  | Diameter | -0.293 | 0.138 | **0.035** |
|  | VPD | 0.321 | 0.051 | **<0.001** |
|  | Competition | 0.402 | 0.232 | 0.101 |
|  | % Competition intrasp. | 0.057 | 0.206 | 0.785 |
|  | Species diversity | -0.115 | 0.183 | 0.538 |
|  | VPD:Competition | 0.194 | 0.059 | **0.001** |
|  | VPD:% Competition intrasp. | -0.218 | 0.061 | **<0.001** |
|  | VPD:Species diversity | -0.137 | 0.058 | **0.018** |
| δ^2^H | Intercept | -35.096 | 2.640 | **<0.001** |
|  | Diameter | 5.422 | 1.597 | **<0.001** |
|  | VPD | 3.129 | 0.566 | **<0.001** |
|  | Competition | -1.998 | 3.371 | 0.561 |
|  | % Competition intrasp. | 2.323 | 3.255 | 0.485 |
|  | Species diversity | 1.152 | 3.052 | 0.710 |
|  | VPD:Competition | -1.174 | 0.654 | 0.073 |
|  | VPD:% Competition intrasp. | 1.882 | 0.680 | **0.006** |
|  | VPD:Species diversity | 1.192 | 0.644 | 0.065 |
| Log(TRW) | Intercept | 1.242 | 0.280 | **<0.001** |
|  | Diameter | -0.055 | 0.150 | 0.713 |
|  | VPD | -0.071 | 0.012 | **<0.001** |
|  | Competition | -0.105 | 0.166 | 0.536 |
|  | % Competition intrasp. | 0.048 | 0.135 | 0.727 |
|  | Species diversity | 0.084 | 0.118 | 0.486 |
|  | VPD:Competition | 0.028 | 0.014 | **0.048** |
|  | VPD:% Competition intrasp. | -0.041 | 0.015 | **0.005** |
|  | VPD:Species diversity | -0.030 | 0.014 | **0.032** |
|  |  |  |  |  |
|  |  |  |  |  |
| **Douglas-fir** | | | | |
| **Response variable** | **Predictor variable** | **Estimate** | **Standard error** | **P-value** |
| δ^13^C | Intercept | -21.923 | 0.312 | **<0.001** |
|  | Diameter | -0.111 | 0.079 | 0.161 |
|  | VPD | 0.219 | 0.020 | **<0.001** |
|  | Competition | -0.204 | 0.141 | 0.164 |
|  | % Competition intrasp. | -0.071 | 0.136 | 0.606 |
|  | Species diversity | -0.006 | 0.121 | 0.962 |
|  | VPD:Competition | 0.021 | 0.021 | 0.337 |
|  | VPD:% Competition intrasp. | 0.024 | 0.021 | 0.258 |
|  | VPD:Species diversity | -0.014 | 0.020 | 0.477 |
| δ^18^O | Intercept | 28.881 | 0.091 | **<0.001** |
|  | Diameter | 0.018 | 0.093 | 0.848 |
|  | VPD | 0.291 | 0.036 | **<0.001** |
|  | Competition | 0.039 | 0.102 | 0.709 |
|  | % Competition intrasp. | -0.141 | 0.102 | 0.182 |
|  | Species diversity | -0.195 | 0.100 | 0.067 |
|  | VPD:Competition | 0.032 | 0.039 | 0.414 |
|  | VPD:% Competition intrasp. | -0.015 | 0.038 | 0.704 |
|  | VPD:Species diversity | -0.048 | 0.037 | 0.195 |
| δ^2^H | Intercept | -44.117 | 2.887 | **<0.001** |
|  | Diameter | -0.091 | 1.564 | 0.954 |
|  | VPD | 3.515 | 0.405 | **<0.001** |
|  | Competition | 2.383 | 3.010 | 0.439 |
|  | % Competition intrasp. | -3.842 | 2.972 | 0.212 |
|  | Species diversity | 2.837 | 2.840 | 0.331 |
|  | VPD:Competition | -0.083 | 0.434 | 0.849 |
|  | VPD:% Competition intrasp. | 0.397 | 0.428 | 0.354 |
|  | VPD:Species diversity | -0.190 | 0.411 | 0.644 |
| Log(TRW) | Intercept | 1.180 | 0.088 | **<0.001** |
|  | Diameter | 0.003 | 0.077 | 0.972 |
|  | VPD | -0.069 | 0.009 | **<0.001** |
|  | Competition | -0.059 | 0.086 | 0.502 |
|  | % Competition intrasp. | -0.052 | 0.085 | 0.547 |
|  | Species diversity | -0.102 | 0.081 | 0.226 |
|  | VPD:Competition | 0.018 | 0.010 | 0.087 |
|  | VPD:% Competition intrasp. | -0.007 | 0.010 | 0.491 |
|  | VPD:Species diversity | -0.002 | 0.010 | 0.845 |

**References**

Allen, R. G., Pereira, L. S., Raes, D., Smith, M., & others. (1998). *Crop evapotranspiration-Guidelines for computing crop water requirements-FAO Irrigation and drainage paper 56* (p. 300). FAO – Food and Agriculture Organization of the United Nations.
