# Supplementary material for: Triple-isotope analysis in tree-ring cellulose suggests only moderate effects of tree species mixture on the climate sensitivity of silver fir and Douglas-fir
Source: Tree Physiol. 2024 Jun 14;44(7):tpae067. doi: 10.1093/treephys/tpae067 (PMC11247184; doi:10.1093/treephys/tpae067)
Supplement: Supplementary_Data_maps_sampling_tpae067 [file supplementary_data_maps_sampling_tpae067.pdf]

Triple-isotope analysis in tree-ring cellulose suggests only moderate effects of tree species mixture on the climate sensitivity of silver fir and Douglas-fir

Justine Charlet de Sauvage, Kerstin Treydte, Matthias Saurer, Mathieu Lévesque

Supplementary Data

Details of the sampling and maps of the study sites. The aerial images are from the Federal Office of Topography swisstopo. The coordinates of the target trees may be offset by several meters from the real position of the target trees due to GPS accuracy (Oregon 750t, Garmin, USA). The distances between target trees and neighbor trees were measured with an accuracy of 0.1 m with a Vertex 4 measuring device (Haglöf, Sweden).

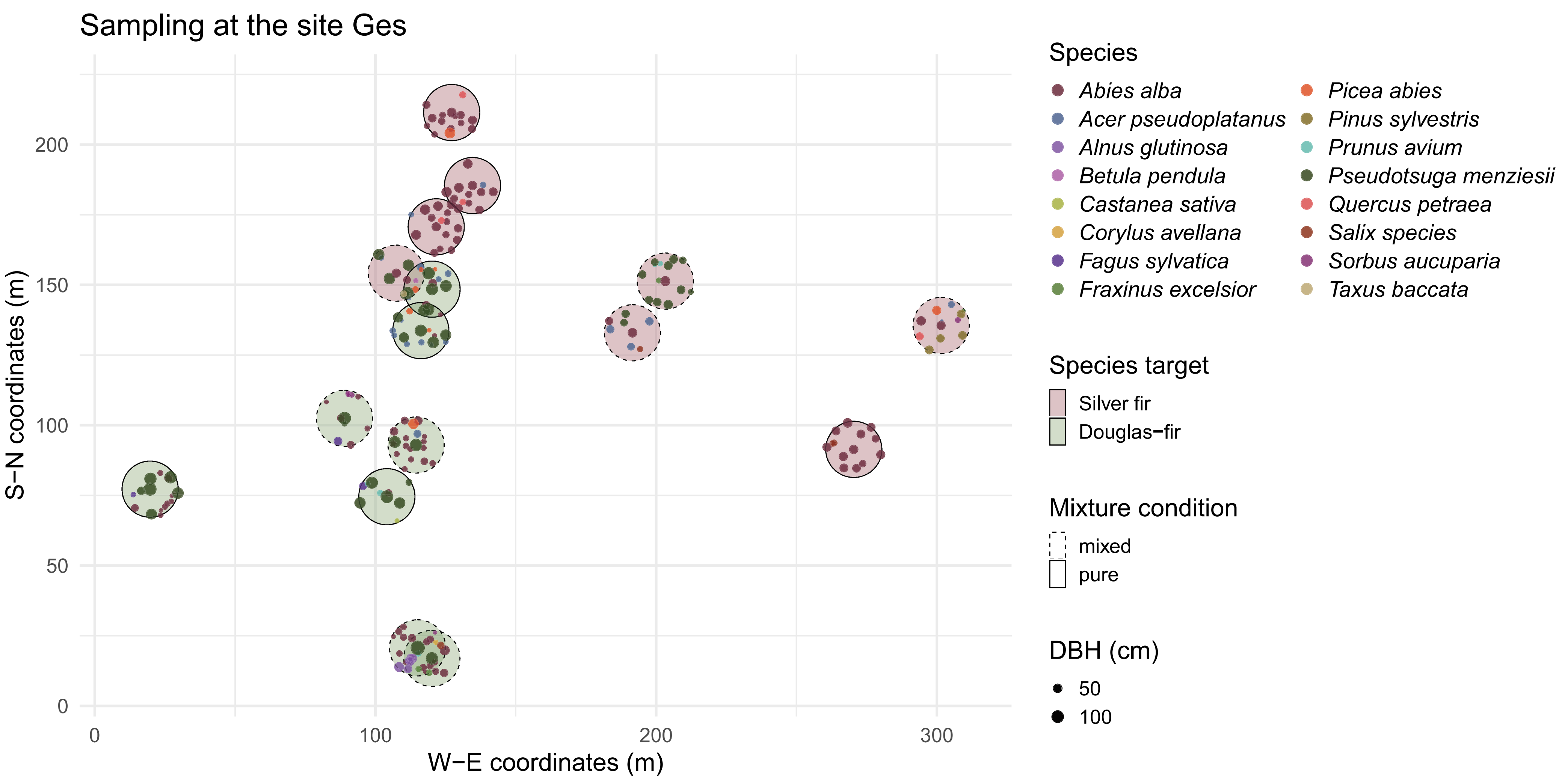
Sampling at the site Ges
S−N coordinates (m)
W−E coordinates (m)
0
50
100
150
200
300
Species
Abies alba
Acer pseudoplatanus
Alnus glutinosa
Betula pendula
Castanea sativa
Corylus avellana
Fagus sylvatica
Fraxinus excelsior
Picea abies
Pinus sylvestris
Prunus avium
Pseudotsuga menziesii
Quercus petraea
Salix species
Sorbus aucuparia
Taxus baccata
Species target
Silver fir
Douglas−fir
Mixture condition
mixed
pure
DBH (cm)
50
100

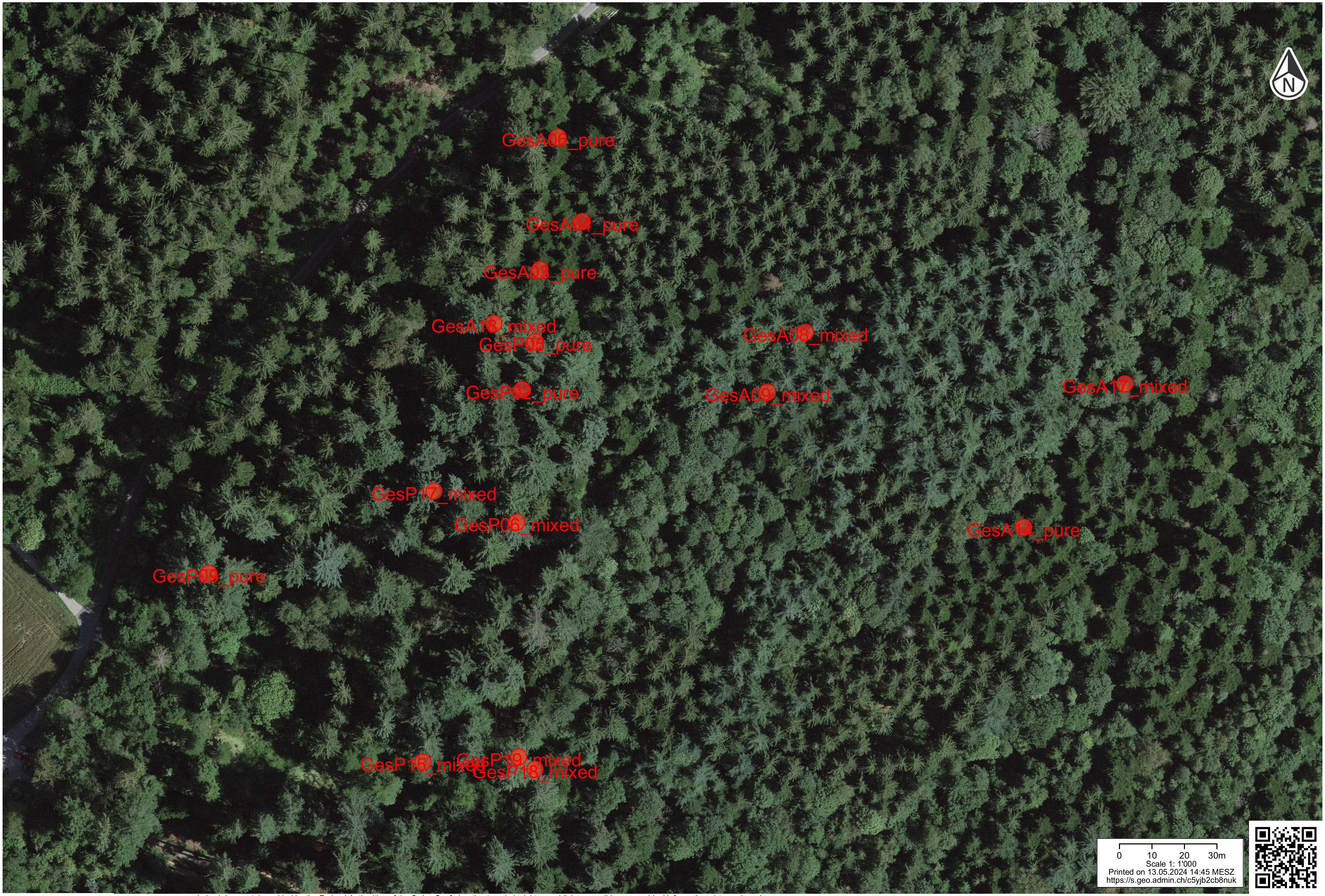

Schweizerische Eidgenossenschaft
Confédération suisse
Confederazione Svizzera
Confederaziun svizra
In collaboration with the cantons

www.geo.admin.ch is a portal provided by the Federal Authorities of the Swiss Confederation to gain insight on publicly accessible geographical information, data and services
Limitation of liability. Although every care has been taken by the Federal Authorities to ensure the accuracy of the information published, no warranty can be given in respect of the accuracy, reliability, up-to-dateness or completeness of this information. Copyright, Swiss federal authorities.
http://www.disclaimer.admin.ch/terms_and_conditions.html
© CNES, Spot Image, swisstopo, NPOC, User local file

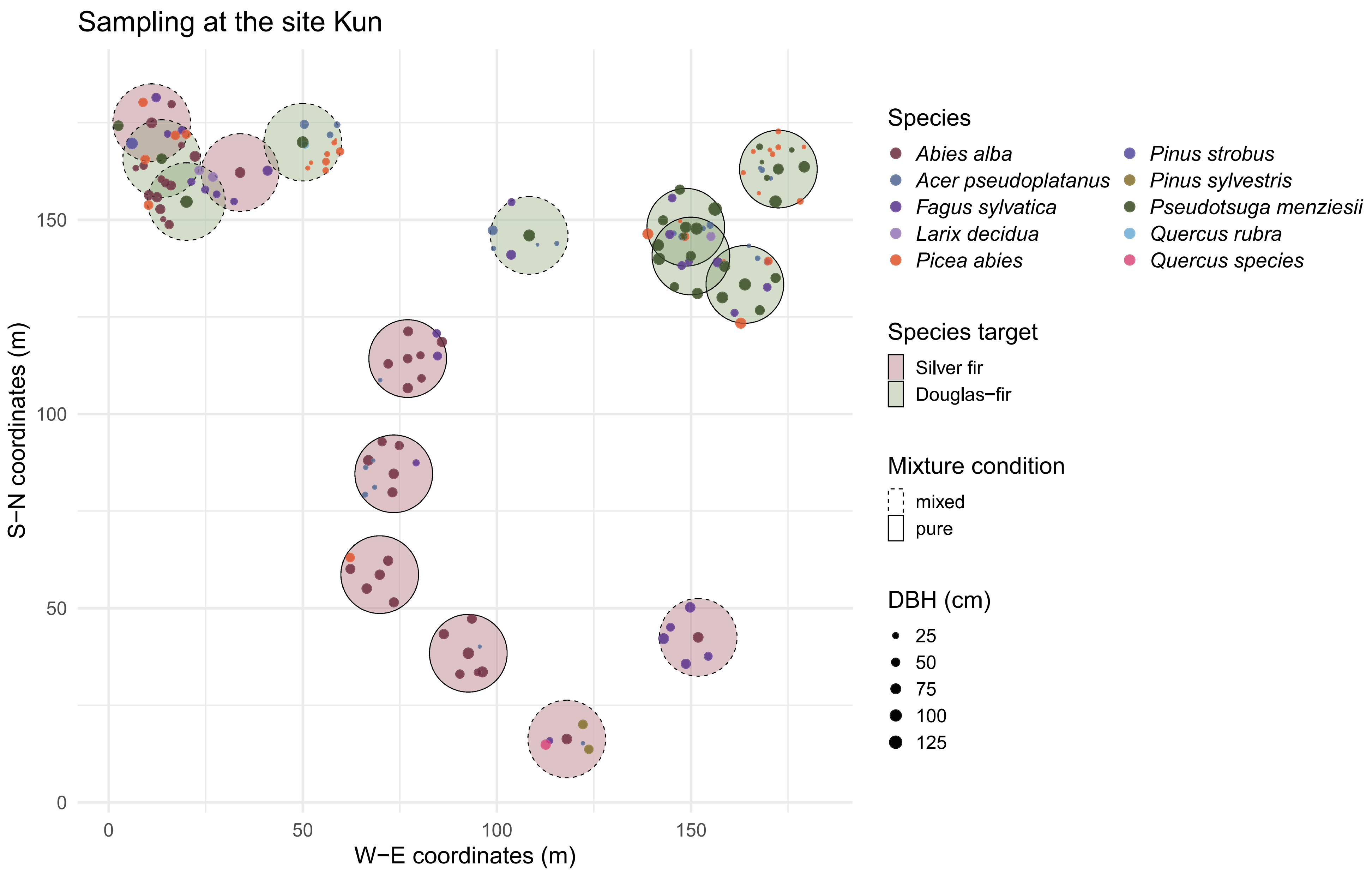

Sampling at the site Kun
S−N coordinates (m)
0
50
100
150
W−E coordinates (m)
Species
Abies alba
Acer pseudoplatanus
Fagus sylvatica
Larix decidua
Picea abies
Pinus strobus
Pinus sylvestris
Pseudotsuga menziesii
Quercus rubra
Quercus species
Species target
Silver fir
Douglas−fir
Mixture condition
mixed
pure
DBH (cm)
25
50
75
100
125

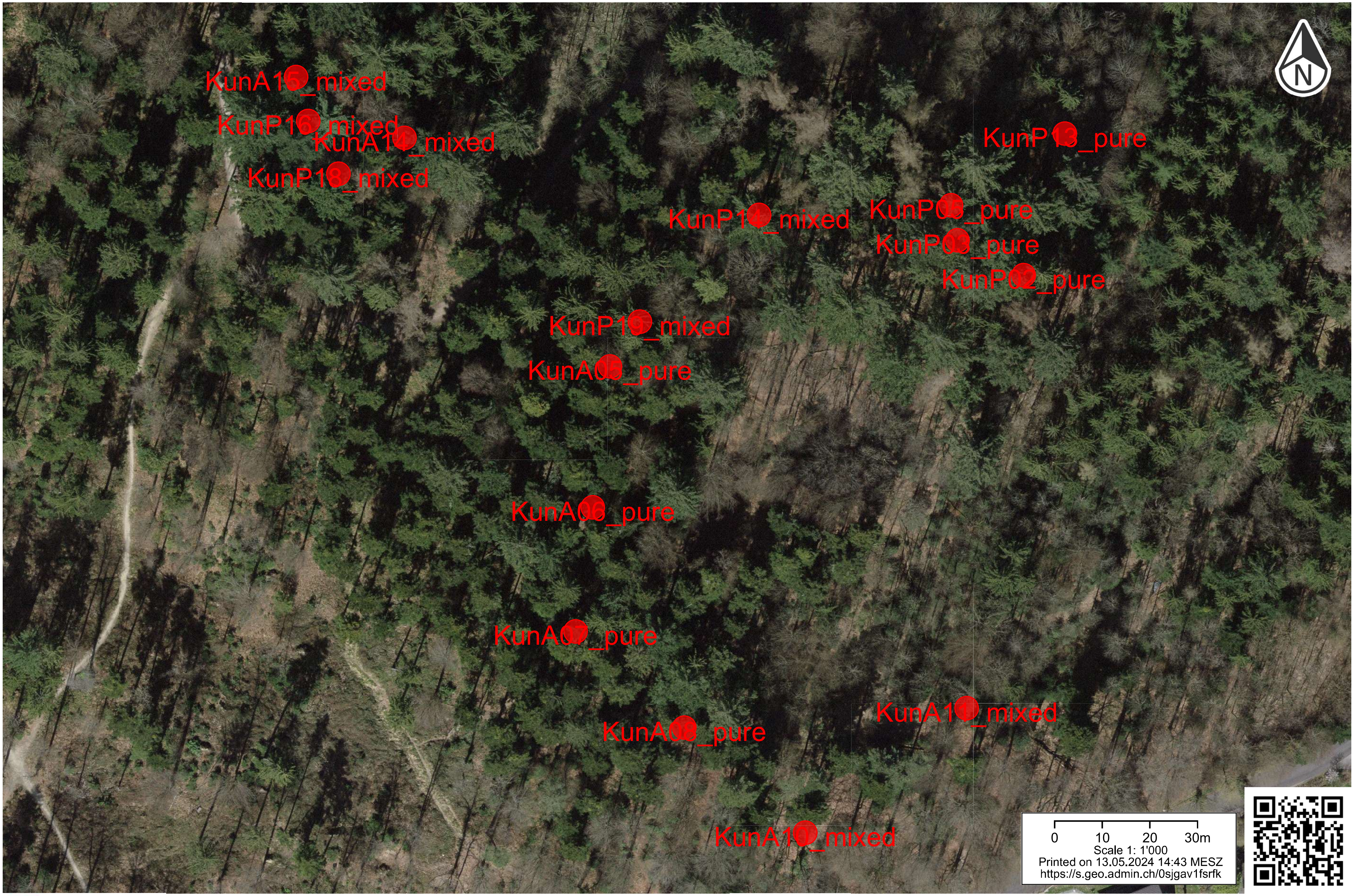

Schweizerische Eidgenossenschaft
Confédération suisse
Confederazione Svizzera
Confederaziun svizra
In collaboration with the cantons

www.geo.admin.ch is a portal provided by the Federal Authorities of the Swiss Confederation to gain insight on publicly accessible geographical information, data and services
Limitation of liability. Although every care has been taken by the Federal Authorities to ensure the accuracy of the information published, no warranty can be given in respect of the accuracy, reliability, up-to-dateness or completeness of this information. Copyright, Swiss federal authorities. http://www.disclaimer.admin.ch/terms_and_conditions.html
© CNES, Spot Image, swisstopo, NPOC, User local file

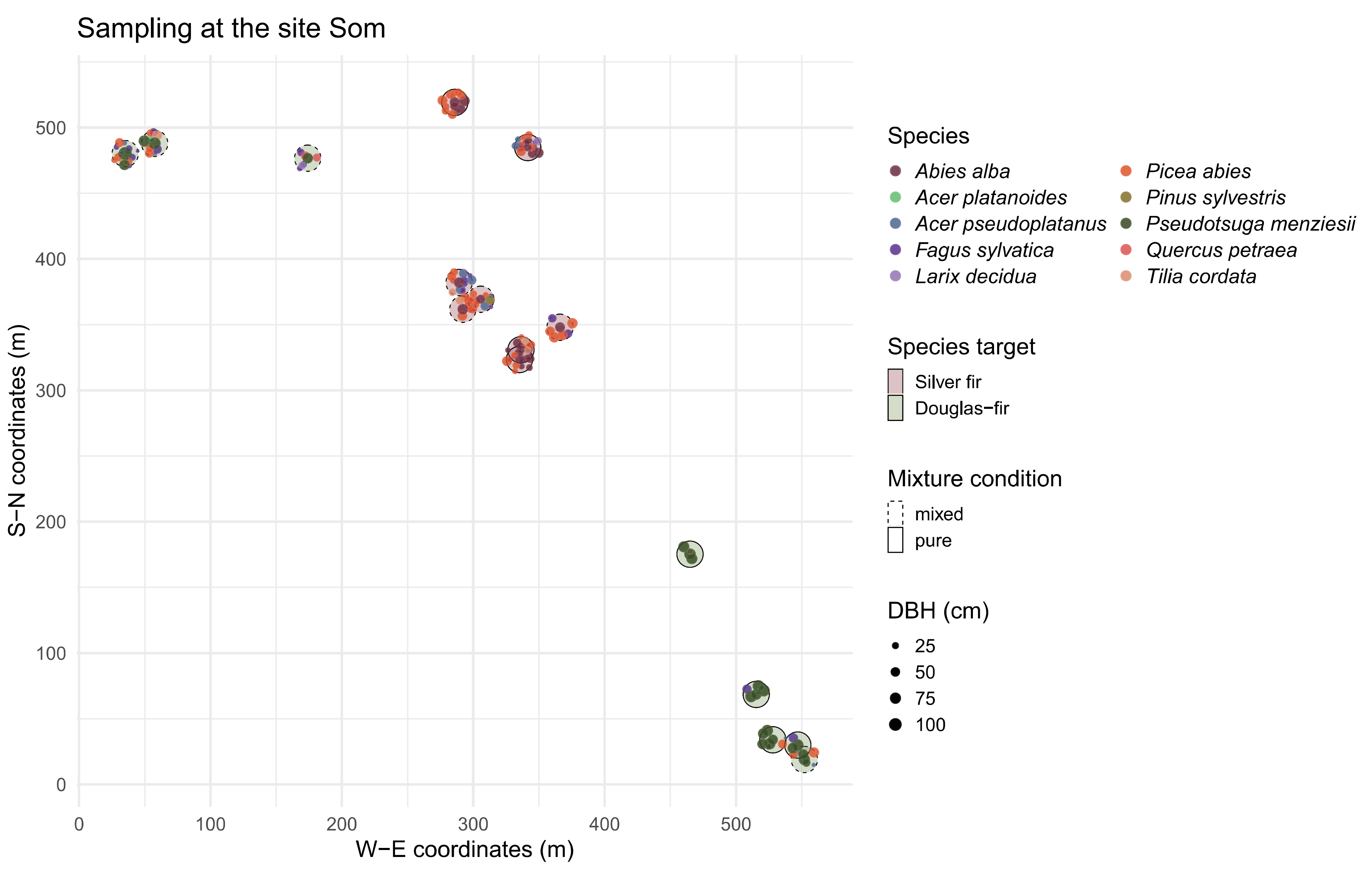
Sampling at the site Som
S−N coordinates (m)
W−E coordinates (m)
0
100
200
300
400
500
Species
Abies alba
Acer platanoides
Acer pseudoplatanus
Fagus sylvatica
Larix decidua
Picea abies
Pinus sylvestris
Pseudotsuga menziesii
Quercus petraea
Tilia cordata
Species target
Silver fir
Douglas−fir
Mixture condition
mixed
pure
DBH (cm)
25
50
75
100

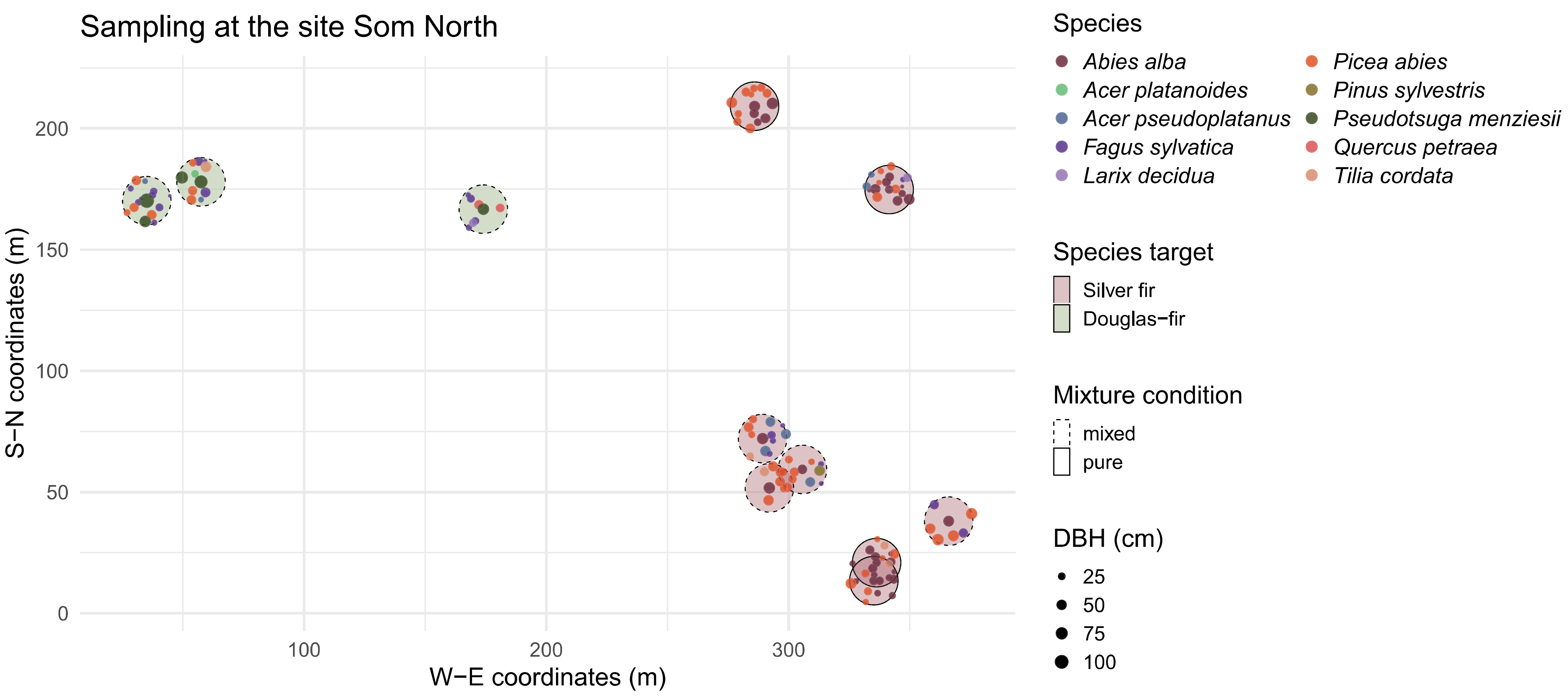

Sampling at the site Som North
S−N coordinates (m)
W−E coordinates (m)
0
50
100
150
200
100
200
300
Species
Abies alba
Acer platanoides
Acer pseudoplatanus
Fagus sylvatica
Larix decidua
Picea abies
Pinus sylvestris
Pseudotsuga menziesii
Quercus petraea
Tilia cordata
Species target
Silver fir
Douglas−fir
Mixture condition
mixed
pure
DBH (cm)
25
50
75
100

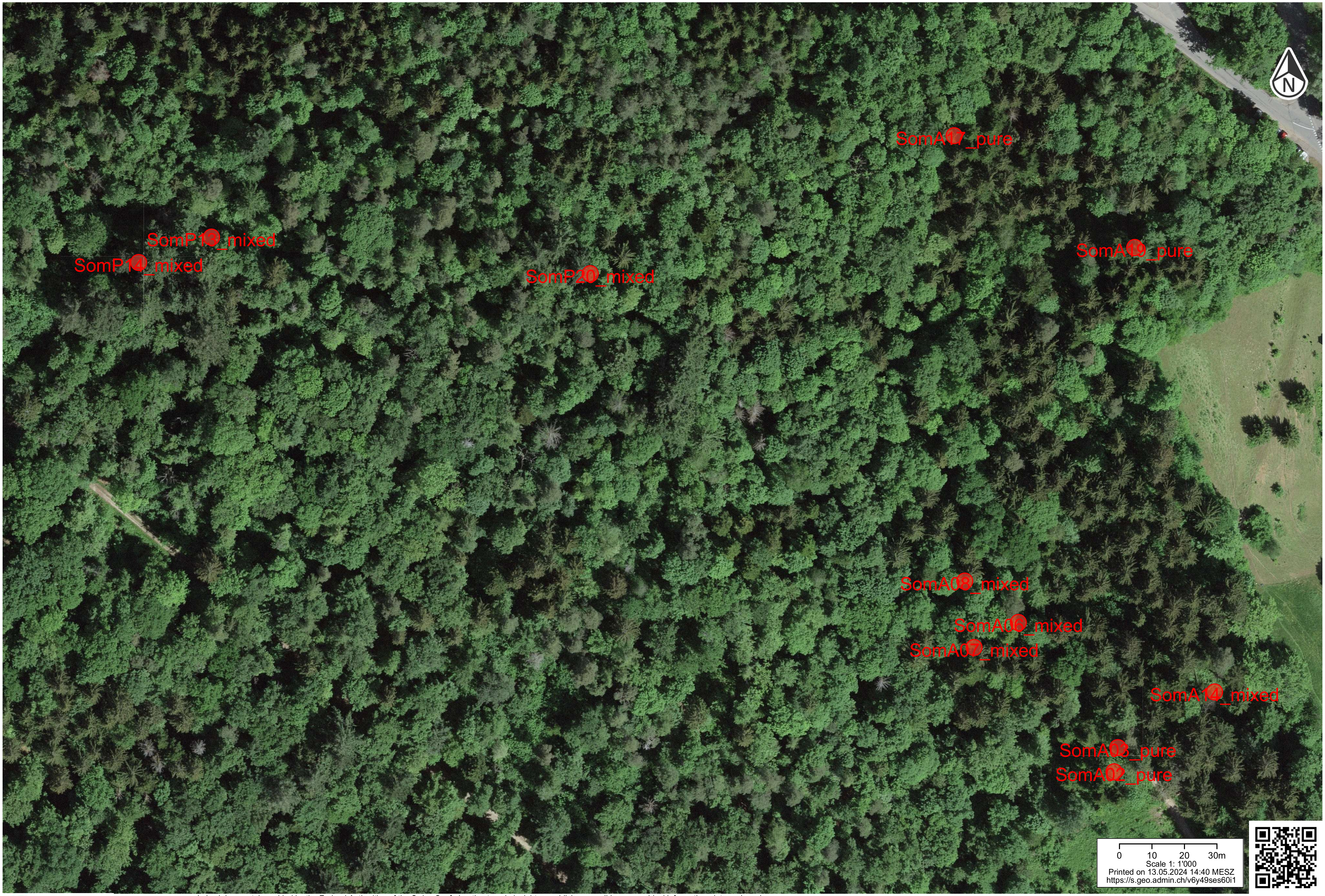

Schweizerische Eidgenossenschaft
Confédération suisse
Confederazione Svizzera
Confederaziun svizra
In collaboration with the cantons

www.geo.admin.ch is a portal provided by the Federal Authorities of the Swiss Confederation to gain insight on publicly accessible geographical information, data and services
Limitation of liability. Although every care has been taken by the Federal Authorities to ensure the accuracy of the information published, no warranty can be given in respect of the accuracy, reliability, up-to-dateness or completeness of this information. Copyright, Swiss federal authorities.
http://www.disclaimer.admin.ch/terms_and_conditions.html
© CNES, Spot Image, swisstopo, NPOC, User local file

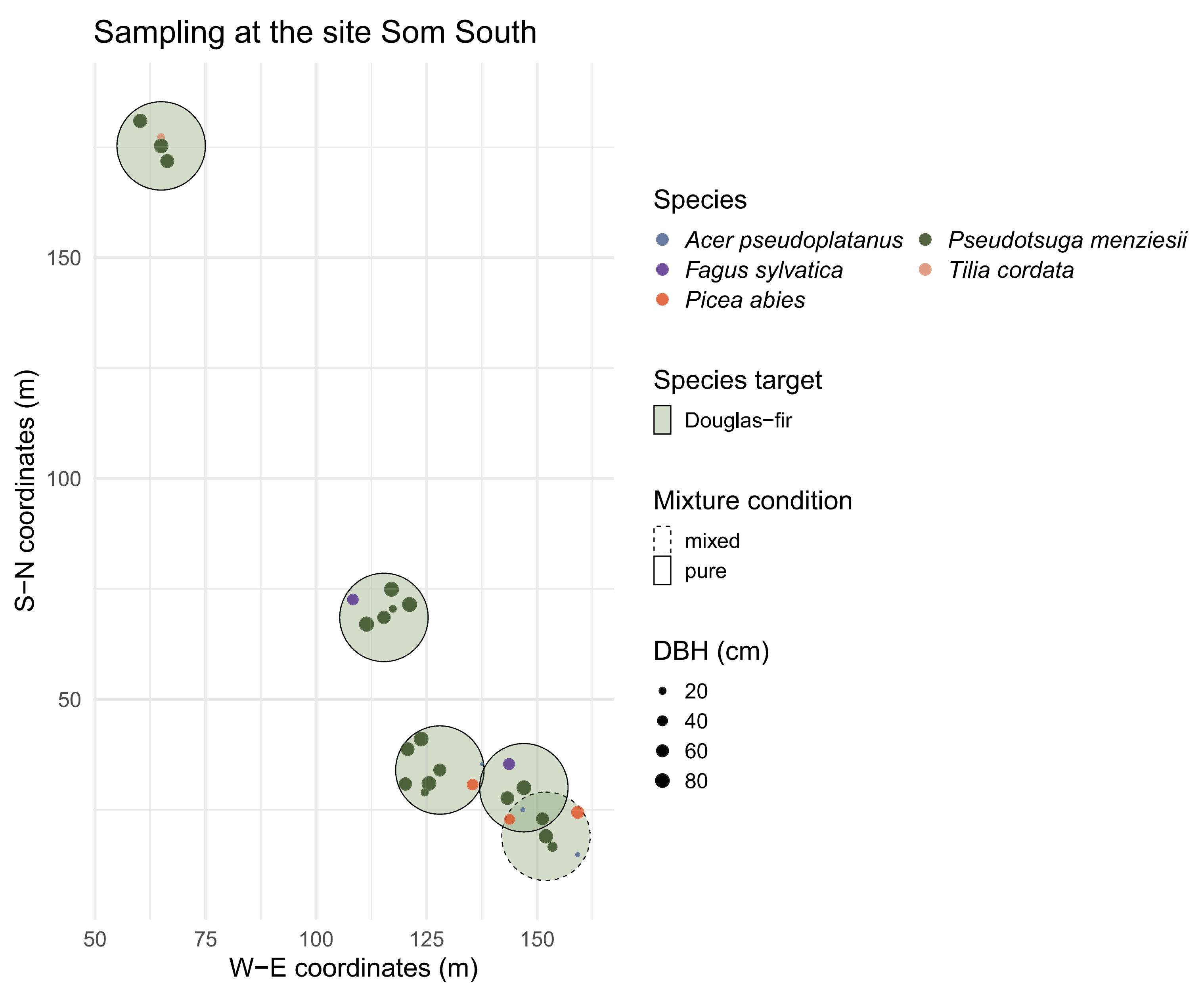
Sampling at the site Som South
S−N coordinates (m)
150
100
50
W−E coordinates (m)
50
75
100
125
150
Species
Acer pseudoplatanus
Fagus sylvatica
Picea abies
Pseudotsuga menziesii
Tilia cordata
Species target
Douglas−fir
Mixture condition
mixed
pure
DBH (cm)
20
40
60
80

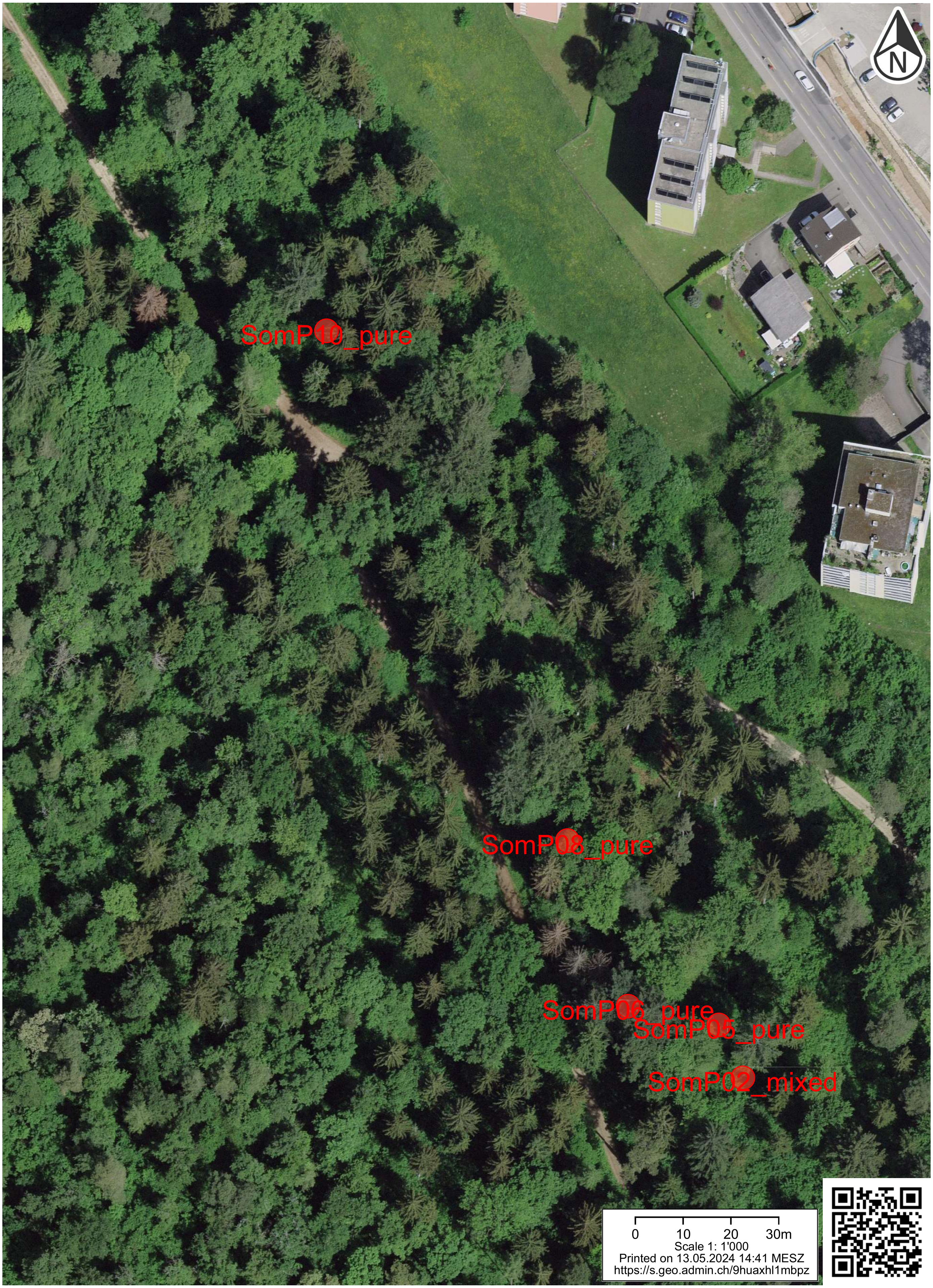

Schweizerische Eidgenossenschaft
Confédération suisse
Confederazione Svizzera
Confederaziun svizra
In collaboration with the cantons

www.geo.admin.ch is a portal provided by the Federal Authorities of the Swiss Confederation to gain insight on publicly accessible geographical information, data and services
Limitation of liability. Although every care has been taken by the Federal Authorities to ensure the accuracy of the information published, no warranty can be given in respect of the accuracy, reliability, up-to-dateness or completeness of this information. Copyright, Swiss federal authorities.
http://www.disclaimer.admin.ch/terms_and_conditions.html
© CNES, Spot Image, swisstopo, NPOC, User local file
